# Yeast enzyme hydrolysis slurry supplementation improves growth, intestinal health, and metabolic responses in juvenile largemouth bass (*Micropterus salmoides*) fed soybean meal-based diets with partial fishmeal replacement

**DOI:** 10.1186/s40104-025-01349-9

**Published:** 2026-03-03

**Authors:** Jun Wen, Xinpeng Wang, Haiqing Wu, Chuyi Cui, Qianyu Zhou, Xue Fu, Shuqing Shen, Shunying Xiao, Yongjun Chen, Shimei Lin, Qinghui Ai, Guangjun Lv, Yuanfa He

**Affiliations:** 1 College of Fisheries, Southwest University, Chongqing, 400715 China; 2 Chongqing Changnuo Biotechnology Co., Ltd., Chongqing, 401520 China; 3 Key Laboratory of Aquaculture Nutrition and Feed (Ministry of Agriculture and Rural Affairs), Ocean University of China, Qingdao, 266005 China

**Keywords:** Gut health, Gut microbiota, Largemouth bass, Metabolomics, Yeast enzyme hydrolysis slurry

## Abstract

**Background:**

Yeast enzyme hydrolysis slurry (YS) has the potential to optimize feed utilization efficiency and improve the health of farmed animals, as it contains abundant bioactive components like small-molecule peptides and amino acids. However, its function and application effects in juvenile largemouth bass (*Micropterus salmoides*) are unclear.

**Methods:**

Three hundred and twenty largemouth bass (8.20 ± 0.05 g) were randomly divided into four groups (4 replicates of 20 fish). Four isonitrogenous (52%) and isolipidic (10%) diets were formulated: FM group (positive control), SBM group (soybean meal replaced 30% of fish meal protein, negative control), and the SBM group supplemented with 1% YS (SBM + 1% YS) and 2% YS (SBM + 2% YS), respectively. After a 56-day feeding period, the fish were assessed for growth, intestinal health, and metabolic regulation-related indices.

**Results:**

Our study found that weight gain rate (*P* = 0.032) and specific growth rate (*P* = 0.030) in the SBM + 1% YS and SBM + 2% YS groups were significantly higher than those in the SBM group. Relative to the SBM group, YS-supplemented groups exhibited marked elevations in intestinal folds, goblet cell numbers, serum acid and alkaline phosphatase activities, catalase and superoxide dismutase activities, as well as the activities of key digestive enzymes (lipase, α-amylase, pepsin, chymotrypsin), accompanied by downregulated mRNA expression of anorexigenic genes cholecystokinin and leptin. Meanwhile, these groups showed significantly lower serum D-lactate, diamine oxidase, lipopolysaccharide levels and malondialdehyde content. The abundance of beneficial genus *Cetobacterium* increased while the abundance of pathogenic genus *Edwardsiella* (*P* = 0.0265) significantly reduced in SBM + 1% YS and SBM comparison groups. Metabolomics analysis revealed that protein digestion and absorption (*P* = 0.0041), and amino acid metabolism pathways (*P* = 0.0052) were significantly enriched in the comparison between SBM + 1%YS and SBM groups. Correlation analysis further indicated that differential metabolites such as arginine and methionine exhibite a strong negative association with *Edwardsiella.*

**Conclusion:**

Yeast enzyme hydrolysis slurry in soybean meal-based diets with partial fishmeal replacement enhanced the antioxidant capacity, reduced intestinal permeability, altered the abundances of intestinal microbiota and associated core metabolites. These positive changes collectively contributed to improved growth performance in largemouth bass.

## Background

Fish meal, characterized by its high protein content, palatability, digestibility, and favorable nutritional profile, is generally considered to be the primary source of protein in formulated diets for aquatic animals [1]. However, constraints in fish meal supply, coupled with escalating costs, have intensified research into alternative protein sources. Plant-based protein ingredients have gained significant interest due to their cost-effectiveness and ready availability [2]. However, the application of plant protein-based feeds in carnivorous fish diets is often limited by inherent challenges; these include imbalanced amino acid profiles and the occurrence of antinutritional factors [3, 4]. Consequently, developing functional additives to enhance the utilization efficiency of plant proteins has emerged as a key research focus.

Yeast-derived products (including yeast hydrolysate, yeast extract, yeast culture, and yeast cell wall extract) have demonstrated potential for improving feed palatability, modulating intestinal microbiota, and enhancing immunity because of their high levels of bioactive components, including nucleotides, small peptides, and B vitamins [5–7]. For instance, supplementation of 1%−5% yeast hydrolysate in plant protein diets enhanced the digestive and antioxidant capacities of the midgut and hindgut, as well as improved intestinal health in juvenile yellow catfish (*Pelteobagrus vachelli* ♂ × *Pelteobagrus fulvidraco* ♀) [8, 9]. Additionally, adding 2% yeast hydrolysate in soybean meal-based diets enhanced the feed utilization and mitigated the adverse effects on liver function and immune responses in pikeperch (*Sander lucioperca*) [10]. The inclusion of 3% yeast extract in fish meal-free diets improved feed quality and growth performance for rainbow trout (*Oncorhynchus mykiss*) [11]. Furthermore, adding yeast cell wall extract to mycotoxin-contaminated feed not only mitigated the toxic effects of the feed and improved growth performance and immune capability but also optimized intestinal health in fish [12, 13]. Similarly, supplementing yeast culture in low-fish meal diets enhanced the intestinal barrier function and modulated the intestinal microbial community in aquatic animals [14, 15]. Yeast enzymatic hydrolysis slurry (YS) is produced from specific fresh *Saccharomyces cerevisiae* through a series of processes: washing, enzymatic hydrolysis for 4 h, low-temperature concentration to a moisture content of 50%, enzyme deactivation and sterilization at 95 °C, cooling to room temperature, packaging into ton barrels, and final storage at room temperature [16, 17]. Compared with products like yeast enzymatic hydrolysis powder, yeast enzymatic hydrolysis slurry has advantages such as lower cost, less loss of functional peptides caused by high temperature, and avoidance of toxic and harmful substances generated by high temperature. The amino acids and small peptides it contains can stimulate the sensory perception of aquatic animals, activate liver function, and promote intestinal repair, thereby enhancing feed palatability, supplementing nutrients, boosting disease resistance, and improving growth performance [18]. However, there are relatively few research reports on yeast enzymatic hydrolysis slurry in aquatic animals.

As an important economic fish species in China, the largemouth bass (*Micropterus salmoides*) achieved an aquaculture output of 938,509 tons in 2024 [19]. Renowned for its delicate flesh and the absence of intermuscular bones, it has become a pillar species in the aquaculture industry [20]. Although the beneficial effects of yeast-based products in largemouth bass have been documented, including improvements in growth performance, muscle quality, immune function, and intestinal health, the application of yeast enzymatic hydrolysis slurry in feed for this species remains understudied [9, 21, 22]. Therefore, this research was designed to assess the influences of supplementing YS in soybean meal-based diets with partial fishmeal replacement on growth performance, intestinal health, and metabolic regulation of largemouth bass. By integrating analysis of physiological indices, 16S rRNA gene sequencing, and untargeted metabolomics, this study further elucidates the potential mechanisms by which YS alleviates the negative effects associated with plant protein utilization.

## Materials and methods

### Animals, experimental design, and diet

The juvenile largemouth bass employed in this experiment were purchased from a single farm (Chongqing Xiaoxiao Agriculture Co., Ltd., China). Before the feeding experiment was initiated, the fish were disinfected and acclimated in 400-L circular tanks for two weeks by filtered city tap water. During acclimation, the fish were fed to satiation twice daily with a commercial diet (Guangzhou Jieda Feed Co., Ltd., China). Following acclimation, a total of 320 healthy fish of consistent size (8.20 ± 0.05 g) were arbitrarily assigned to 16 blue circular fiberglass tanks, each having a capacity of 210 L. There were 20 fish per culture tank, with four replicate tanks assigned to each of the four experimental groups. The rearing period lasted for 8 weeks. Artificial fish feeding at 08:30 and 17:30 daily. A photoperiod of 12L:12D was maintained. Culture water, which was filtered city tap water, was partially exchanged daily, and waste feed and feces were removed via siphoning. Throughout the experimental period, water quality parameters were regulated as follows: dissolved oxygen > 6 mg/L, total ammonia nitrogen concentration < 0.2 mg/L, and water temperature in the range of 28–30 °C.

Four isonitrogenous (52% crude protein) and isolipidic (10% crude lipid) diets were formulated as follows: fish meal group (FM, positive control), soybean meal group (soybean meal replaced 30% of fish meal, SBM, negative control), and the soybean meal group supplemented with 1% YS (SBM + 1% YS) and 2% YS (SBM + 2% YS), respectively. Peruvian fishmeal was used as the fishmeal resource, and dehulled soybean was adopted as the soybean meal. The processing of soybeans involves cleaning and crushing, followed by n-hexane extraction, desolventization, drying, crushing, and classification to obtain the finished dehulled soybean meal. The experimental feed formulations are shown in Table 1. In line with the specified formula, all protein-based raw materials were sieved using a 60-mesh screen to ensure accurate weighing of each component. A step-by-step mixing approach was employed to add ingredients in smaller amounts to the feed mixture. Add 30% water and oil sources to the mixture, then thoroughly mix them in an Automatic S-type Single-paddle Trough Mixer (Model: CT-C-200, Jiangyin Xiangda Machinery Manufacturing Co., Ltd., China). The mixture was then processed into 2 mm pellets via cold extrusion using a feed pelletizer (Model: SG-YPYS-76, Xiamen Xinyunfa Machinery Equipment Factory, China) to produce 2 mm diameter pellets. The feed prepared in this experiment was stored in a refrigerator at −20 °C for later use, and subsequent to feed preparation, the proximate composition of each experiment feed was determined using the AOAC method [23].

**Table 1:** Composition of experimental diets and nutrient content

Ingredients, %	Groups			
	FM	SBM	SBM + 1%YS	SBM + 2%YS
Peruvian fishmeal^a^	42.00	29.40	29.40	29.40
Chicken meal^a^	15.00	15.00	15.00	15.00
Plasma protein powder^a^	5.00	5.00	5.00	5.00
Rapeseed meal^a^	3.80	3.80	3.80	3.80
Cottonseed protein concentrate^a^	8.40	8.40	8.40	8.40
Dehulled soybean meal^a^	0.00	16.97	16.97	16.97
Yeast enzyme hydrolysis slurry^b^	0.00	0.00	1.00	2.00
Wheat flour	10.00	10.00	10.00	10.00
Fish oil	2.00	2.00	2.00	2.00
Soybean oil	1.50	2.00	2.00	2.00
Microcrystalline cellulose	7.28	2.46	1.66	0.86
Ca(H_2_PO_4_)_2_	1.50	1.50	1.50	1.50
Choline chloride	0.50	0.50	0.50	0.50
Vitamin C	0.10	0.10	0.10	0.10
Mineral and vitamin premixes	2.00	2.00	2.00	2.00
Ethoxyquinoline	0.05	0.05	0.05	0.05
Lysine^c^	0.00	0.14	0.14	0.14
Methionine^c^	0.17	0.28	0.28	0.28
Non-essential amino acids	0.70	0.40	0.20	0.00
Proximate composition				
Crude protein	52.99	52.54	52.30	52.70
Crude lipid	10.06	9.85	9.91	9.94
Crude ash	15.01	13.92	13.89	14.08
Moisture	10.19	10.16	10.40	10.53

^a^Purchased from Chongqing Citico Biotechnology Co., Ltd. (Chongqing, China). Peruvian fishmeal: crude protein 65.22%, crude lipid 10.37%. Chicken meal: crude protein 62.77%, crude lipid 12.50%. Plasma protein powder: crude protein 87.01%, crude lipid 1.00%. Rapeseed meal: crude protein 36.00%, crude lipid 1.97%. Cottonseed protein concentrate: crude protein 60.00%, crude lipid 4.61%. Dehulled soybean meal: crude protein 48.43%, crude lipid 3.18%

^b^Purchased from Chongqing Changnuo Biotechnology Co., Ltd. (Chongqing, China), crude protein 22.30%, moisture 59.00%, crude ash 2.60, amino acid nitrogen 2.29%, mannan 3.01%

^c^Purchased from Shanghai Sanjie Biotechnology Co., Ltd. (Shanghai, China). Levorotatory amino acid with purity ≥ 98%

### Sample collection

Before sampling, the experimental fish need to be fasted for 24 h and anesthetized using a 0.1% MS-222 solution (Sigma-Aldrich, USA) at the time of sampling. Each fish was individually weighed and counted for the calculation of growth performance. Subsequently, a random sample of nine fish per tank was taken for comprehensive analysis, including body length measurement, blood collection from the caudal vein, and dissection. The weights of the viscera, liver, and intestine were measured to evaluate the physiological parameters. The freshly drawn blood was kept at 4 °C for 24 h, then centrifuged at 3,500 r/min for 15 min. The supernatant was collected and stored at −80 °C for subsequent analysis of serum biochemical indicators and intestinal permeability assessment. The foregut, midgut, and hindgut of 2 fish per tank were sampled and preserved in 4% paraformaldehyde solution to be used in follow-up hematoxylin and eosin (H&E) staining. Two fish were chosen at random from each experimental tank, and their hindguts were pooled into a composite sample for antioxidant enzyme activity assay; two foreguts were collected from each tank and mixed into one sample for digestive enzyme activity assay. In addition, the hindguts of four fish selected from each tank were pooled into composite samples for gut microbiota assay, and the hindguts of another four fish were collected and pooled into composite samples for metabolomics analysis. Four fish brains were randomly selected from each tank, pooled into composite samples, and transferred to RNase-free centrifuge tubes produced by LABBSELECT for gene expression analysis. Except for tissues designated for histological processing, the samples were quickly frozen and then stored at −80 °C for future use.

### Intestinal histological analysis

Pre-fixed intestinal tissues underwent gradual dehydration through an ethanol gradient, followed by paraffin embedding and sectioning at a thickness of 4 μm. After sectioning, H&E staining was performed on the tissues. Histopathological features and goblet cell counts were examined using an optical microscope (Eclipse Ti-E, Japan) coupled with an image acquisition system (NIS Elements, Japan). Twenty intestinal segments were selected from each slice to determine the fold and muscular layer thickness. Intestinal fold width (IFW), intestinal fold height (IFH), muscular layer thickness (MLT), and goblet cell (GC) numbers were measured by Image J software (NIH, USA).

### Serum biochemical parameter analysis

Serum acid phosphatase (ACP, #A060-2-2), alkaline phosphatase (AKP, #A059-2-2), aspartate transaminase (AST, #C010-2-1), and alanine transaminase (ALT, #C009-2-1) were measured to assess immune activity. Serum LPS, DAO (#A088-3-1), and D-Lac (#A019-3-1) were measured to analyze intestinal permeabilities. The detection procedures and calculations were conducted strictly following the manufacturer's protocols (Nanjing Jiancheng Bioengineering Institute, Nanjing, China). Serum LPS (YJ858127) level was determined using a fish-specific ELISA kit (Shanghai Enzyme-linked Biotechnology Co., Ltd., Shanghai, China).

### Detection of intestinal digestive enzyme and antioxidant enzyme activities

Chymotrypsin (#A080-3-1), α-amylase (#C016-1-2), lipase (#A054-2-1), and pepsin (#A080-1-1) in the foregut were measured to evaluate intestinal digestive capacity. Total antioxidant capacity (T-AOC, #A015-2-1), glutathione peroxidase (GSH-PX, #A005-1), catalase (CAT, #A007-1-1), malondialdehyde (MDA, #A003-1-2), and superoxide dismutase (SOD, #A001-3-2) were assayed to reflect antioxidant capacity. All assays were conducted in accordance with the instructions of the commercial kits from Nanjing Jiancheng.

### Intestinal microbiota analysis

Intestinal DNA from FM, SBM, and SBM + 1% YS groups was obtained using the HiPure DNA Kit (Magen, Guangzhou, China). DNA concentration and integrity were assessed via Qubit 3.0 Fluorometer and 2% agarose gel electrophoresis, respectively. For the bacterial 16S rRNA genes, the amplification process targeting their V3–V4 hypervariable regions was carried out with the use of specific primers. (338F: 5′-ACTCCTACGGGAGGCAGCAG-3′; 806R: 5′-GGACTACHVGGGTWTCTAAT-3′). Libraries were constructed and quality-controlled. The Illumina NovaSeq 6000 platform (PE250 mode) was used to sequence the qualified libraries.

The Illumina platform (Gene Denovo Biotechnology Co., Ltd., Guangzhou, China) was employed for paired-end sequencing. Operational taxonomic units (OTUs) were formed by clustering high-quality sequences at a 97% similarity threshold via Uparse v9.2.64. Alpha diversity indices (ACE, Chao1, Simpson, and Shannon) were performed in QIIME 1.9.1. Beta diversity analysis utilizing Bray–Curtis distance metrics, including principal coordinates analysis (PCoA), was conducted. Taxonomic abundance was visualized in Krona 2.6. Stacked bar plots of community composition were generated using the ggplot2 package (v2.2.1) in R. Welch's *t*-test (Vegan package v2.5.3 in R) was applied for inter-group differential abundance analysis.

### Untargeted metabolomics analysis

Intestinal tissue samples (100 mg) from FM, SBM, and SBM + 1% YS groups were homogenized for metabolite extraction. Supernatants and QC samples were analyzed. Equipped with a HILIC column, an Agilent 1290 Infinity UHPLC system was used for the separation. A mass spectrometer of the AB TripleTOF 6600 model was used to gather primary and secondary mass spectra. ProteoWizard (v3.0.6428) was used to convert the raw data into the mzML format. With subsequent procedures involving peak alignment, retention time calibration, and peak area retrieval with XCMS Online (v3.7.1). To enhance metabolic coverage, positive and negative ionization modes were employed. Untargeted metabolomic analysis was entrusted to Gene Denovo Biotechnology Co., Ltd. (Guangzhou, China).

Principal component analysis (PCA) was performed using the gmodels package in R. Orthogonal partial least squares discriminant analysis (OPLS-DA) was conducted using the ropls package, and the reliability of the model was verified through cross-validation and permutation tests. Differential metabolites were identified through the integration of OPLS-DA VIP values and univariate *t*-test *P*-values (threshold: VIP ≥ 1.5 and *P* < 0.05). Volcano plots visualize fold-change trends of differential metabolites. Metabolites were annotated with KEGG compound IDs (C_id), and Omicsmart generated KEGG enrichment bubble plots.

### Feeding-related gene expression analysis

Following the extraction of total RNA from pooled brain tissues with RNAiso Plus reagent (TaKaRa, Japan), RNA concentration and purity were subsequently assessed with a NanoDrop 2000 ultra-micro spectrophotometer. Reverse transcription kit (RR092A; TaKaRa, Japan) was used to perform reverse transcription reactions and synthesize cDNA. The design and synthesis of all primers were conducted by Sangon Biotech (Guangzhou) Co., Ltd., with specific primer details listed in Table 2. Real-time quantitative PCR was performed using the TB Green Premix Ex Taq II kit (RR820A; TaKaRa, Japan). A CFX96 Touch™ Real-Time Fluorescence Quantitative PCR Detection System (Bio-Rad, Hercules, CA, USA) was used to carry out amplification reactions. The qPCR reaction protocol comprised the following steps: an initial pre-denaturation stage at 95 °C lasting 2 min, after which 40 cycles were run, and each cycle included a 5 s denaturation step at 95 °C and a 1 min annealing/extension step at 60 °C. The relative expression levels of the target genes were quantitatively analyzed using the 2^−ΔΔCT^ method [24], with the *ef1α* gene serving as the internal reference.

**Table 2:** Real-time quantitative PCR primers for feeding-related genes in largemouth bass

Genes	Nucleotide sequence (5'→3')	Accession No.
*ef1α*	F: GTTGCTGCTGGTGTTGGTGAG R: GAAACGCTTCTGGCTGTAAGG	XM_038724777.1
*cck*	F: TAAAGGGAAGTCACGGCTCATAC R: CGGTTATTCTCAACAGACCCTGA	XM_038724067.1
*lep*	F: GACTCTCAGCCCACCTTCTG R: GGTAACCCGTCAGCGAAGAG	XM_038706036.1
*npy*	F: ACTCTGGGGTTCCTGCTTTG R: GTACTTGGCTAGCTCGTCCG	XM_038724667.1

*ef1α* Elongation factor 1-alpha, *cck* Cholecystokinin, *lep* Leptin, *npy* Neuropeptide Y

### Calculation and statistical analysis

The formulae involved in this experiment are as follows:

- $$\mathrm{Weight}\;\mathrm{gain}\;\mathrm{rate}\;(\mathrm{WGR})=100\times\lbrack(\mathrm{final}\;\mathrm{weight}\;-\;\mathrm{initial}\;\mathrm{weight})/\mathrm{initial}\;\mathrm{weight}\rbrack.$$
- $$\mathrm{Specific}\;\mathrm{growth}\;\mathrm{rate}\;(\mathrm{SGR})=100\times\lbrack(\ln\;\mathrm{final}\;\mathrm{weight}-\ln\;\mathrm{initial}\;\mathrm{weight})/56\;(\mathrm d)\rbrack.$$
- $$\mathrm{Feeding}\;\mathrm{ratio}\;(\mathrm{FR})=100\times\mathrm{feed}\;\mathrm{intake}/\lbrack(\mathrm{final}\;\mathrm{weight}\;+\;\mathrm{initial}\;\mathrm{weight})/2\times56\;(\mathrm d)\rbrack.\;$$
- $$\mathrm{Protein}\;\mathrm{efficiency}\;\mathrm{rate}\;(\mathrm{PER})=(\mathrm{final}\;\mathrm{weight}-\mathrm{initial}\;\mathrm{weight})/\mathrm{protein}\;\mathrm{intake}.\;$$
- $$\mathrm{Feed}\;\mathrm{conversion}\;\mathrm{ratio}\;(\mathrm{FCR})=(\mathrm{final}\;\mathrm{weight}-\mathrm{initial}\;\mathrm{weight})/\mathrm{feed}\;\mathrm{intake}.$$
- $$\mathrm{Viscera}\;\mathrm{somatic}\;\mathrm{index}\;(\mathrm{VSI})=100\times(\mathrm{visceral}\;\mathrm{weight}/\mathrm{fish}\;\mathrm{weight}).$$
- $$\mathrm{Hepatopancreas}\;\mathrm{somatic}\;\mathrm{index}\;(\mathrm{HSI})=100\times(\mathrm{liver}\;\mathrm{weight}/\mathrm{fish}\;\mathrm{weight}).\;$$
- $$\mathrm{Intestine}\;\mathrm{somatic}\;\mathrm{index}\;(\mathrm{ISI})=100\times(\mathrm{intestinal}\;\mathrm{weight}/\mathrm{fish}\;\mathrm{weight}).\;$$
- $$\mathrm{Condition}\;\mathrm{factor}(\mathrm{CF})=100\times\lbrack\mathrm{final}\;\mathrm{weight}/{(\mathrm{body}\;\mathrm{length})}^3\rbrack.$$

SPSS 27.0 was utilized to perform one-way ANOVA. This step was intended to examine the existence of significant inter-group differences. Results featuring *P* values less than 0.05 were deemed to have statistical significance; subsequent pairwise comparisons were carried out via Duncan’s post-hoc test. All data are shown as “mean ± standard error of the mean (SEM)”. Visual graphics were produced with GraphPad Prism 9.0 (GraphPad Software, Inc., USA).

## Results

### Growth performance and morphometric indices

Among all experimental groups, the SBM group exhibited the lowest FBW, WGR, and SGR values (Table 3). These values in the experimental groups, SBM+1% YS and SBM+2% YS, were notably higher compared to the SBM group (*P* = 0.037, 0.032, 0.030) but were similar to those of the FM group (*P* = 0.106, 0.0976, 0.0958). Additionally, no statistically significant differences were observed in FR, FCR, and PER in any group (*P* = 0.401, 0.573, 0.579). The SBM group exhibited the highest CF value, showing a significant difference from that observed in the FM group (*P* = 0.034). However, the SBM group showed the lowest VSI (*P* < 0.001) and HIS (*P* < 0.001) values, which differed significantly from those of other groups. ISI showed no significant variation in any group (*P* = 0.211).

**Table 3:** Effects of dietary YS on growth performance and morphometric indices of largemouth bass

Items	Groups			
	FM	SBM	SBM + 1%YS	SBM + 2%YS
FBW, g	40.27 ± 0.92^ab^	38.82 ± 0.84^a^	43.12 ± 1.18^b^	43.40 ± 1.46^b^
WGR, %	390.84 ± 11.10^ab^	373.69 ± 10.15^a^	426.42 ± 14.36^b^	429.73 ± 17.36^b^
SGR, %/d	283.96 ± 3.99^ab^	277.63 ± 3.76^a^	296.40 ± 4.87^b^	297.33 ± 5.88^b^
FR, %	2.77 ± 0.09	2.86 ± 0.03	2.79 ± 0.04	2.91 ± 0.07
FCR	1.19 ± 0.06	1.24 ± 0.02	1.16 ± 0.03	1.20 ± 0.05
PER	1.69 ± 0.08	1.62 ± 0.02	1.74 ± 0.04	1.68 ± 0.03
CF, g/cm^3^	2.18 ± 0.04^a^	2.30 ± 0.03^b^	2.25 ± 0.02^ab^	2.24 ± 0.02^ab^
VSI, %	7.67 ± 0.22^c^	6.22 ± 0.10^a^	6.81 ± 0.12^b^	6.70 ± 0.12^b^
HIS, %	2.32 ± 0.08^c^	1.19 ± 0.05^a^	1.58 ± 0.05^b^	1.64 ± 0.06^b^
ISI, %	0.80 ± 0.01	0.76 ± 0.01	0.77 ± 0.01	0.77 ± 0.01

The values of growth performances are mean ± SEM (*n* = 4), and the morphological indicators are mean ± SEM (*n* = 9). Different lowercase letters in the same row indicate statistical differences (*P* < 0.05). *FBW* Final body weight, *WGR* Weight gain rate, *SGR* Specific growth rate, *FR* Feeding rate, *FCR* Feed conversion ratio, *PER* Protein efficiency ratio, *CF* Condition factor, *VSI* Viscerosomatic index, *HSI* Hepatosomatic index, *ISI* Intestinal somatic index

### Intestinal histomorphology analysis

**Fig. 1:**
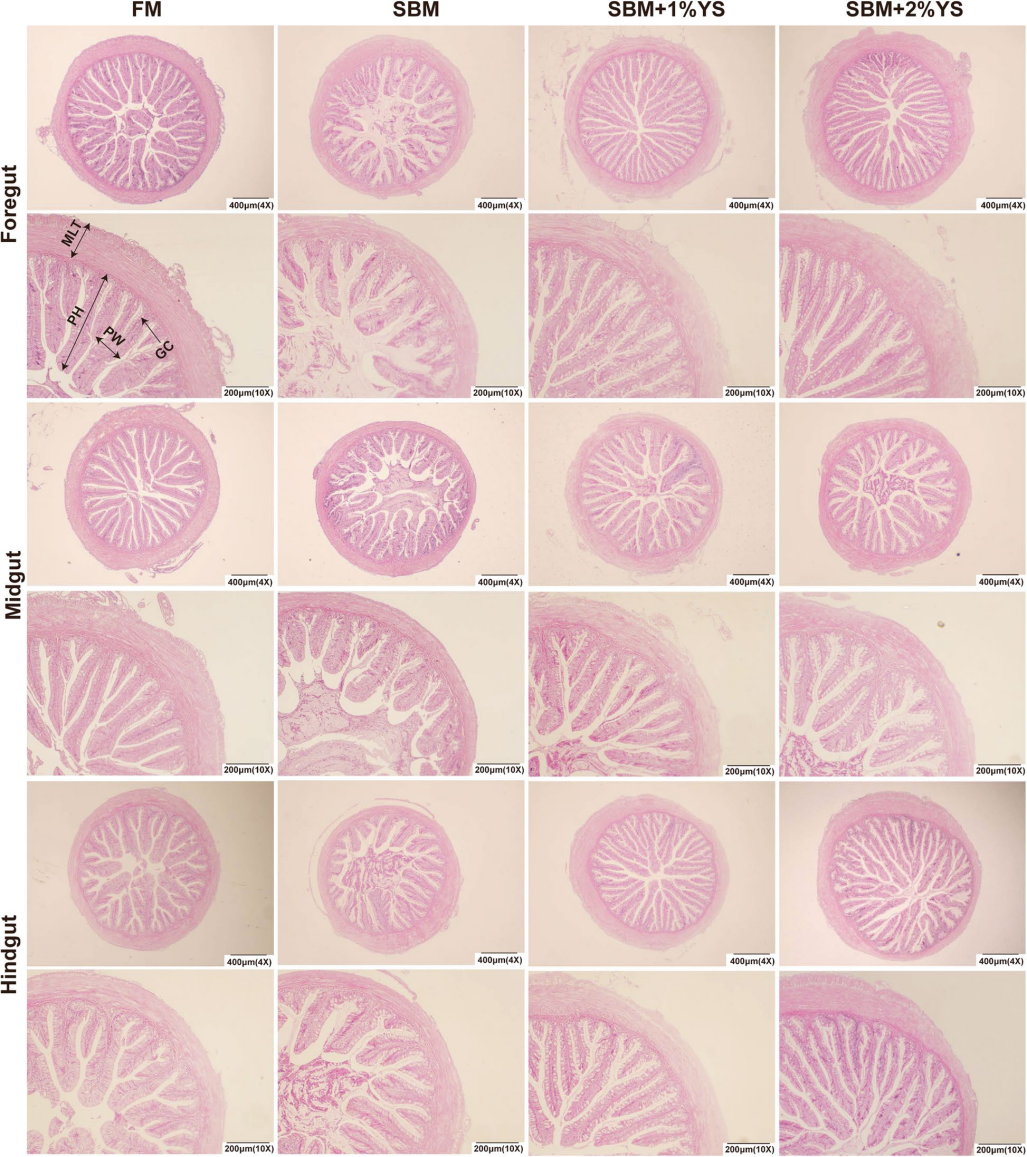
Hematoxylin–Eosin staining of different intestinal segments in juvenile largemouth bass

**Fig. 2:**
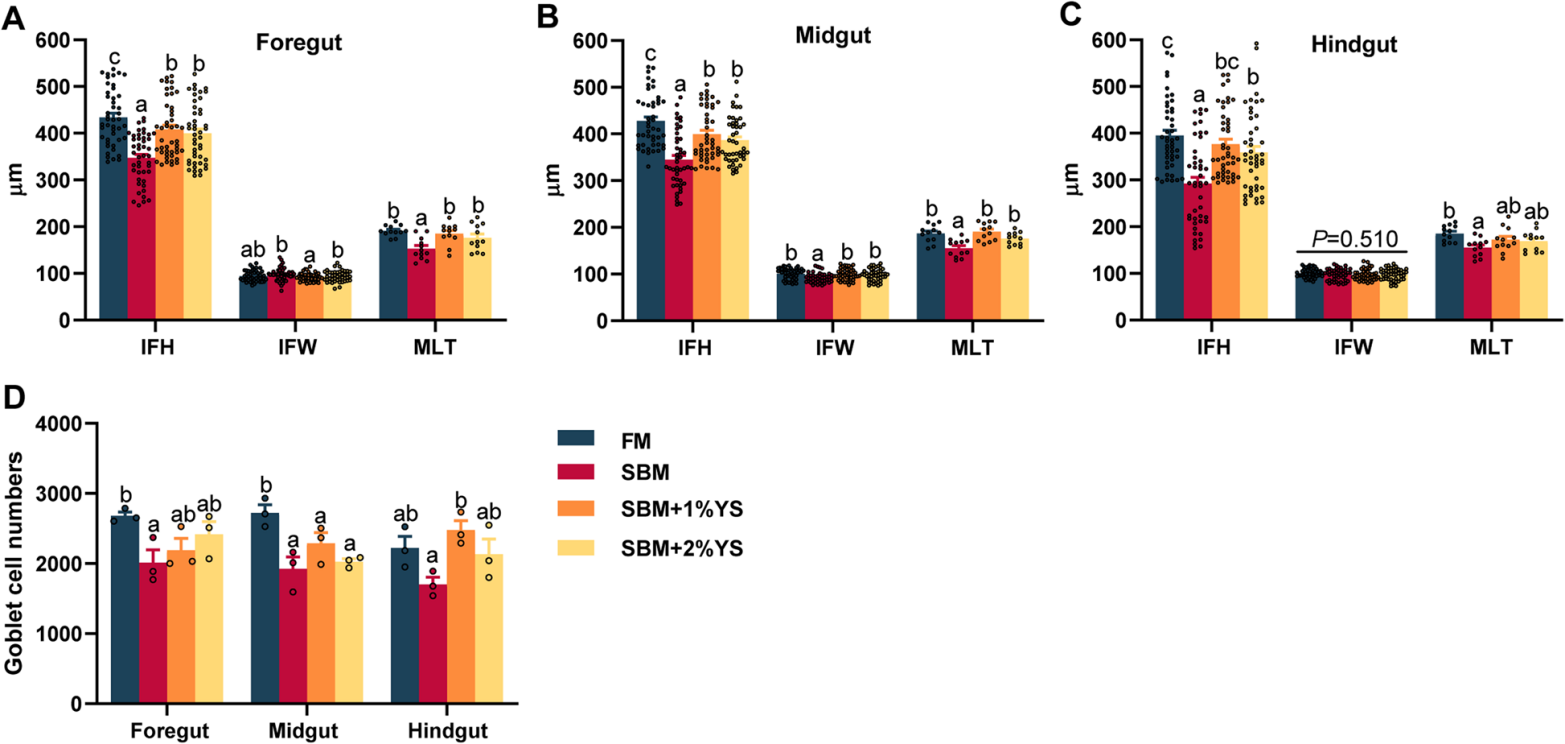
Intestinal histomorphometric measurement in the different intestinal segments of four groups. **A** Intestinal fold height (IFH). **B** Intestinal fold width (IFW). **C** Muscular layer thickness (MLT). **D** Goblet cell numbers (GC). Values are expressed as mean ± SEM (*n* = 4). Columns with different letters represent significantly different (*P* < 0.05)

In the foregut, the SBM group exhibited the lowest IFH, MLT, and GC numbers among all groups (Figs. 1 and 2). For midgut indices, IFH (*P* < 0.001), IFW (*P* = 0.005), and MLT (*P* < 0.001) in the SBM group were notably lower than those in all other groups, while the numbers of GC in the FM group were notably higher than other groups (*P* = 0.011). In the hindgut, the SBM group exhibited the lowest IFH, MLT, and GC numbers. Among the indices, IFH showed a significant reduction relative to other groups (*P* < 0.001), while no statistically significant disparity in IFW was detected when comparing the various groups (*P* = 0.510).

### Serum biochemical analysis

**Fig. 3:**
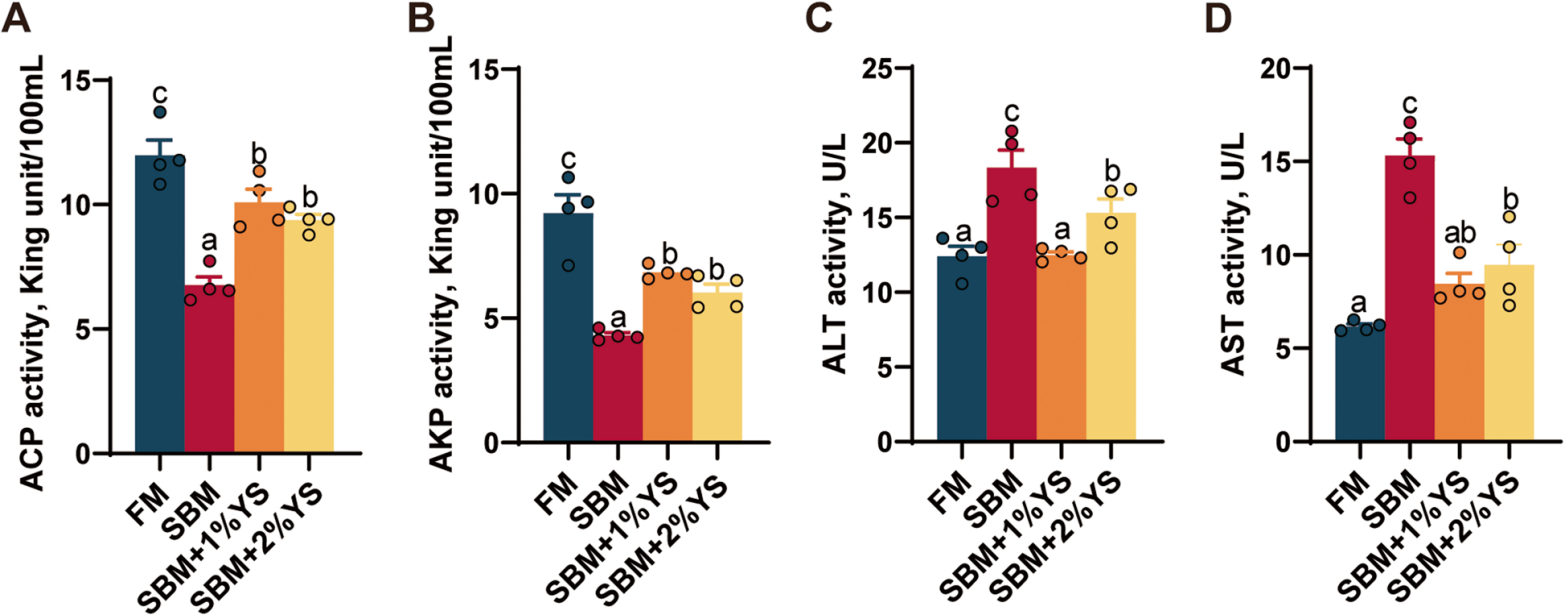
Serum biochemistry indices of juvenile largemouth bass fed with different diets. **A** Acid phosphatase (ACP). **B** Alkaline phosphatase (AKP). **C** Alanine aminotransferase (ALT). **D** Aspartate aminotransferase (AST). Values are expressed as mean ± SEM (*n* = 4). Results with significant differences were marked with different letters (*P* < 0.05)

The SBM group showed significantly lower ACP (*P* = 0.001) and AKP (*P* < 0.001, Fig. 3) activities than all other groups. However, the SBM group exhibited notably higher ALT (*P* < 0.001) and AST (*P* < 0.001) activities than other groups. Moreover, the activities of ALT (*P* = 0.9065) and AST (*P* = 0.0508) in the FM group were comparable to those in the SBM + 1%YS group.

### Intestinal permeability analysis

**Fig. 4:**
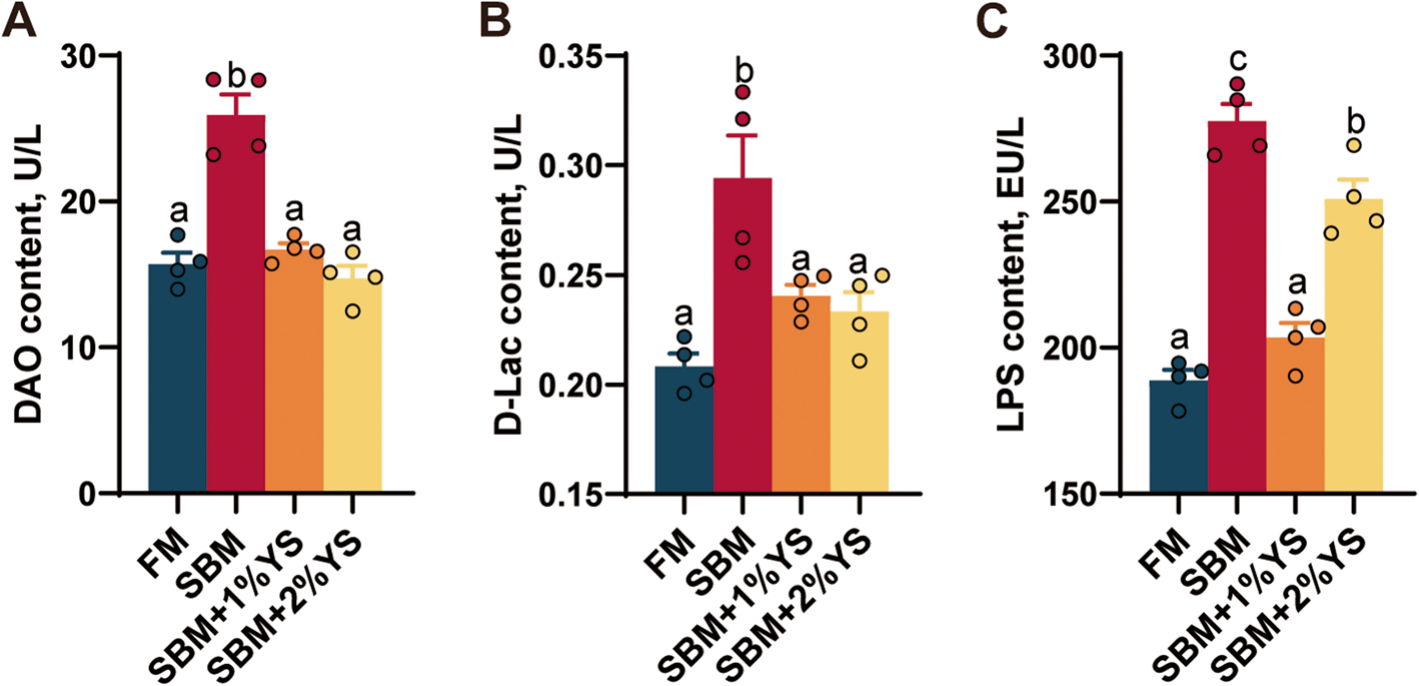
Intestinal permeability parameters of juvenile largemouth bass fed with different diets*.* **A** Diamine oxidase (DAO). **B** D-Lactate (D-Lac). **C** Lipopolysaccharide (LPS). Values are expressed as mean ± SEM (*n* = 4). Results with significant differences were marked with different letters (*P* < 0.05)

Levels of DAO (*P* < 0.001), D-Lac (*P* = 0.001), and LPS (*P* < 0.001) were highest in the SBM group, significantly exceeding those in the FM group and the groups supplemented with varying proportions of yeast enzyme hydrolysis slurry (Fig. 4).

### Intestinal antioxidant enzyme activity analysis

**Fig. 5:**
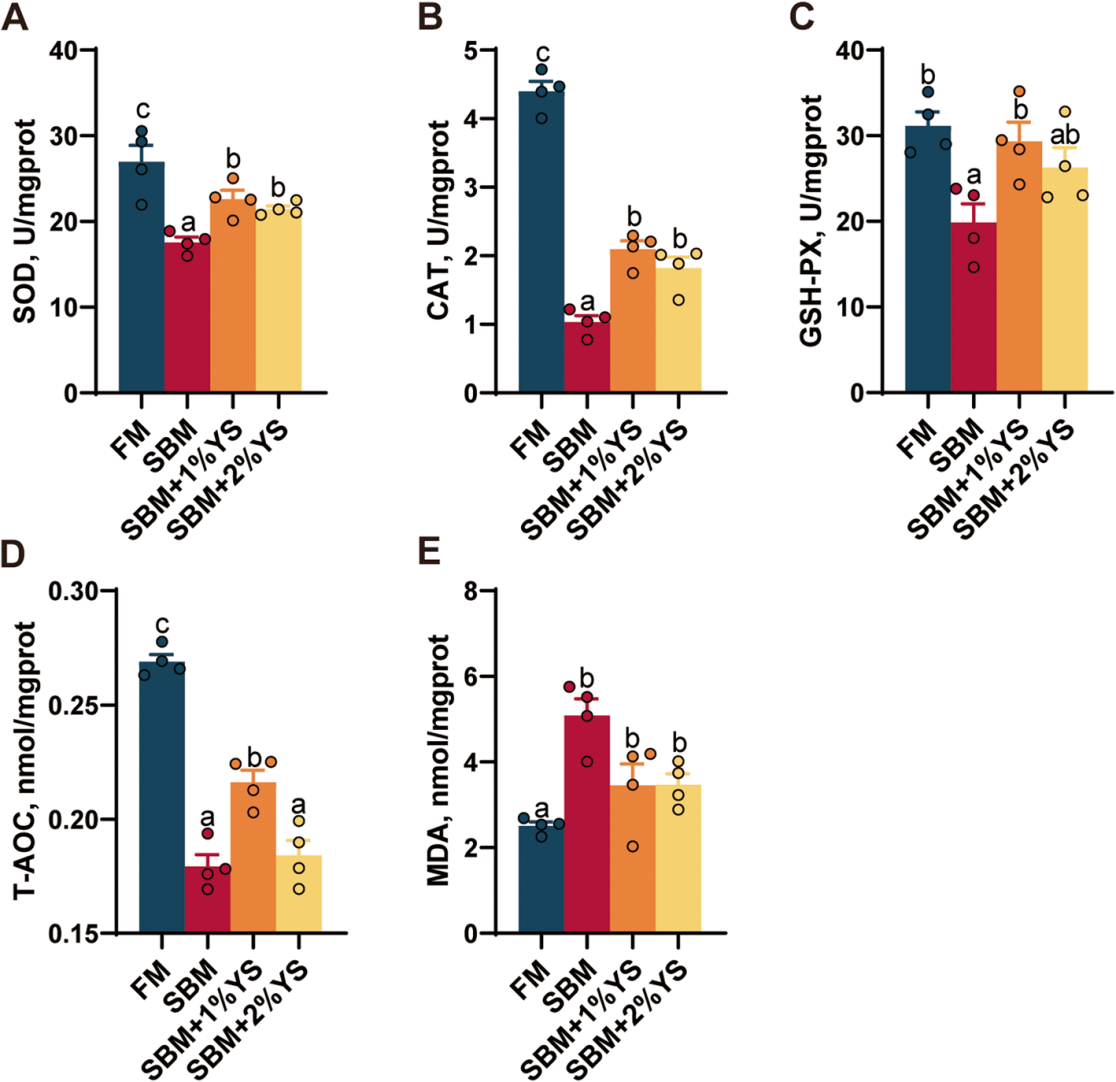
Antioxidant enzyme activity index of the intestine of juvenile largemouth bass fed with different diets. **A** Superoxide dismutase (SOD). **B** Catalase (CAT). **C** Glutathione peroxidase (GSH-Px). **D** Total antioxidant capacity (T-AOC). **E** Malondialdehyde (MDA). Values are expressed as mean ± SEM (*n* = 4). Results with significant differences were marked with different letters (*P* < 0.05)

The FM group had the highest levels of T-AOC, CAT, SOD, and GSH-Px, and the lowest level of MDA (Fig. 5). Furthermore, compared with the SBM group, the catalase activity (*P* < 0.001) and superoxide dismutase activity (*P* = 0.005) in the 1% YS group were significantly increased, while the malondialdehyde level (*P* = 0.0419) was significantly decreased. Similarly, in the 2% YS group, the catalase activity (*P* = 0.0052) and superoxide dismutase activity (*P* = 0.0016) were significantly increased, and the malondialdehyde level (*P* = 0.0127) was significantly decreased.

### Intestinal digestive enzyme activity analysis

**Fig. 6:**
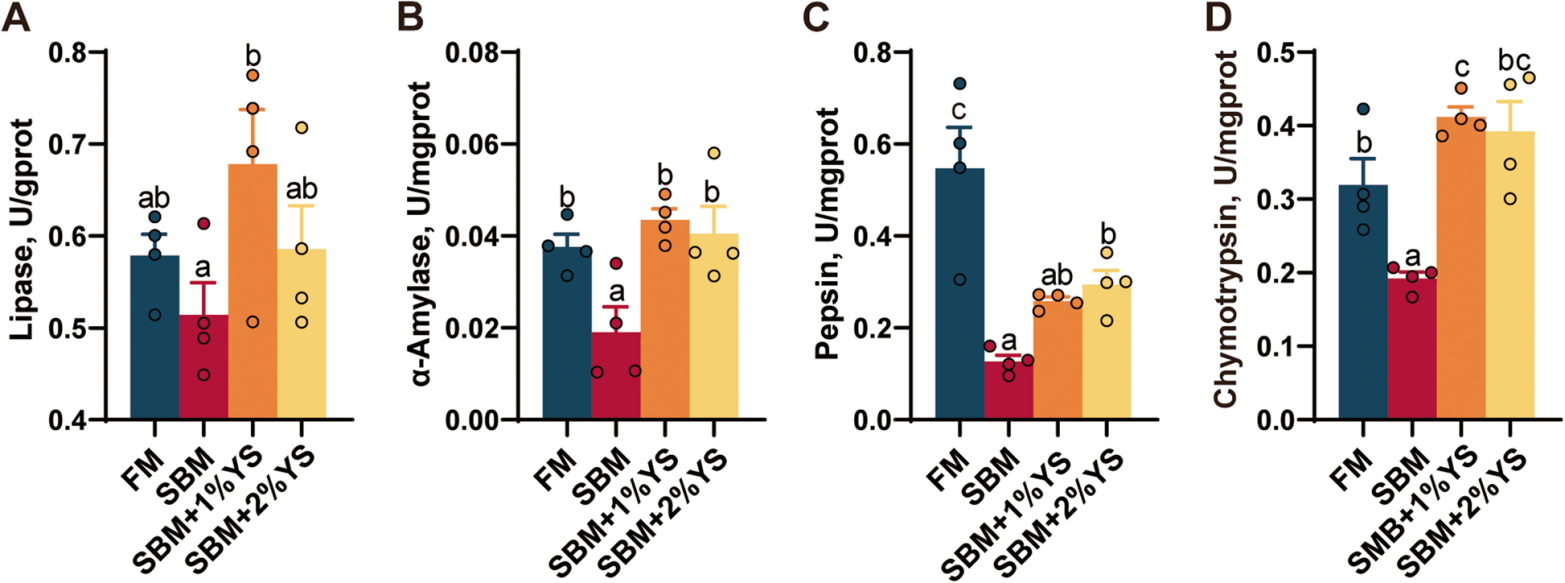
Intestinal digestive enzyme activity of juvenile largemouth bass fed with different diets. **A** Lipase. **B** α-Amylase (α-AMS). **C** Pepsin. **D** Chymotrypsin. Values are expressed as mean ± SEM (*n* = 4). Results with significant differences were marked with different letters (*P* < 0.05)

The SBM group had the lowest activities of all digestive enzymes (Fig. 6). Compared to the SBM group, YS supplementation elevated all enzyme activities (lipase, α-amylase, pepsin, and chymotrypsin). Specifically, the activities of α-amylase (*P* = 0.0068) and chymotrypsin (*P* < 0.001) in the 1% YS group, and the activities of α-amylase (*P* = 0.039) and chymotrypsin (*P* = 0.0029) in the 2% YS group were significantly increased.

### Expression of appetite-regulating genes

**Fig. 7:**
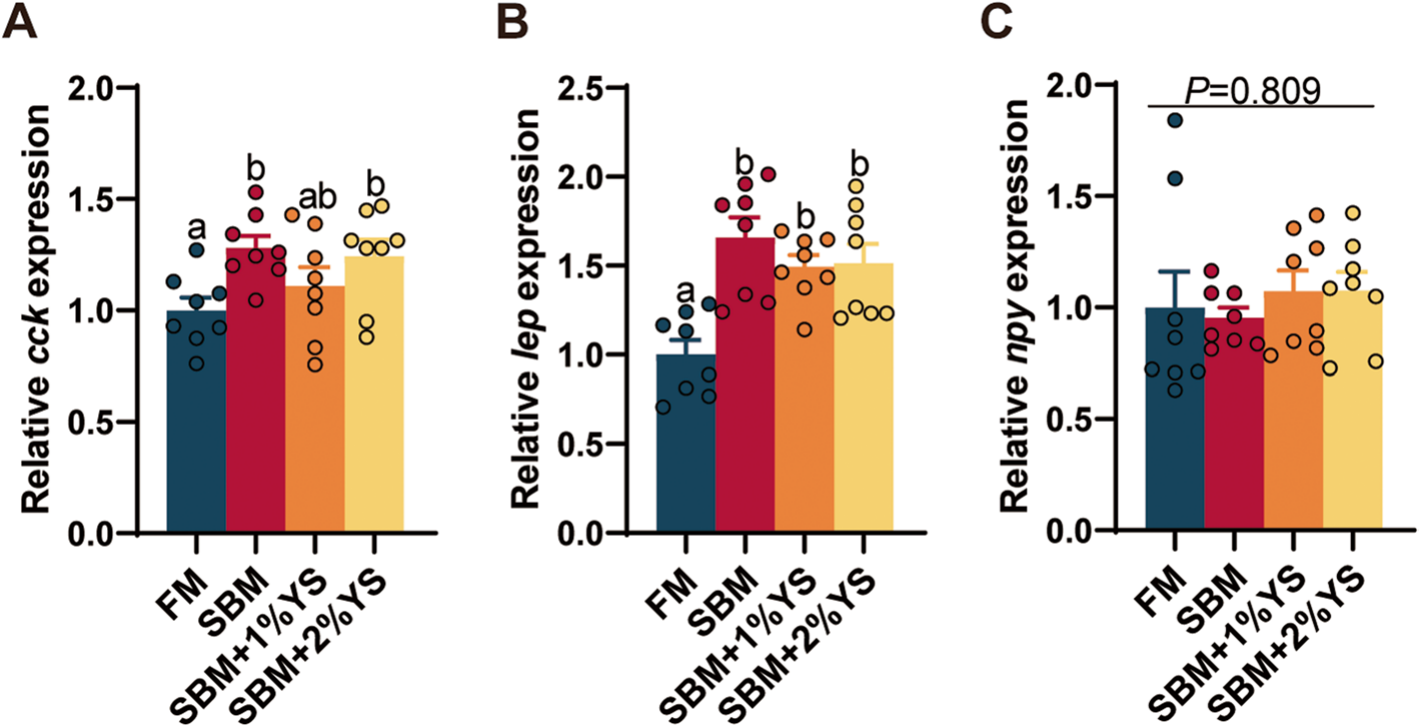
Expression of appetite-regulating hormone and neuropeptide genes of juvenile largemouth bass fed with different diets. **A** Cholecystokinin (*cck*). **B** Leptin (*lep*). **C** Neuropeptide Y (*npy*). Values are expressed as mean ± SEM (*n* = 4). Results with significant differences were marked with different letters (*P* < 0.05)

The mRNA expression levels of the anorexigenic factors *cck* and *lep* reached their highest levels in the SBM group, but were downregulated following YS supplementation (Fig. 7). Conversely, the expression of orexigenic neuropeptides was lowest in the SBM group, whereas YS supplementation elevated their expression beyond levels observed in the FM group (*P* = 0.6992).

### Intestinal microbiota analysis

**Fig. 8:**
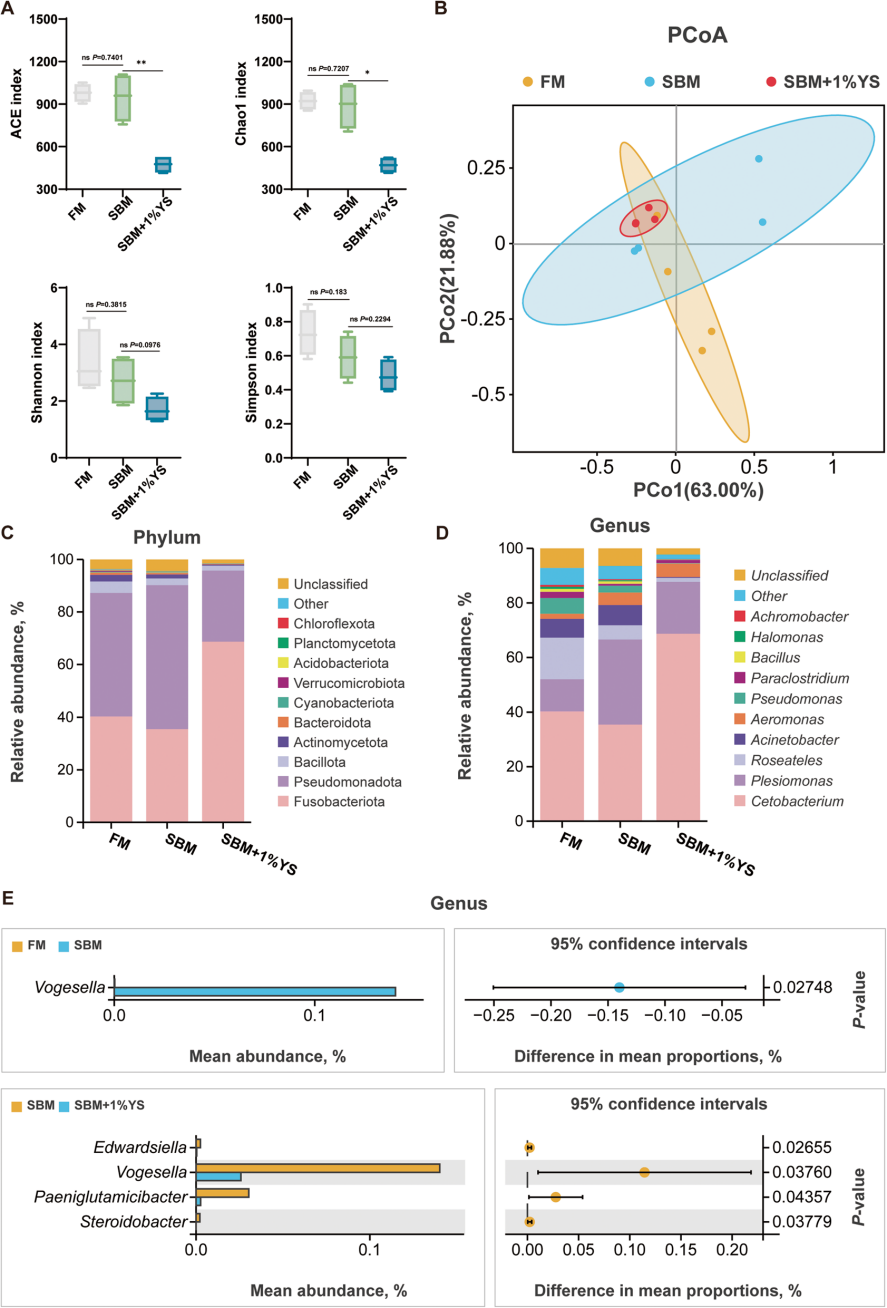
The degree of difference among the three groups was shown for the intestinal microbiota (*n* = 4). **A** Effects of three groups on the intestinal microbiota α diversity of juvenile largemouth bass. **B** Principal component analysis. The *x*-axis represents the first principal component, and the *y*-axis represents the second principal component. The different colored ellipses represent 95% confidence intervals. **C** Histogram of intestinal microbial species classification at the phylum level in the three groups. **D** Histogram of intestinal microbial species classification at the genus level in the three groups. **E** Welch's *t*-test at the genus level

Based on the comprehensive analysis of growth performance, histological morphology, and physiological and biochemical indicators as described above, the intestinal samples of the FM, SBM, and SBM + 1%YS groups were selected for microbial diversity analysis. The SBM + 1%YS group exhibited the lowest ACE, Chao1, Shannon, and Simpson indices, with ACE (*P* = 0.0022) and Chao1 (*P* = 0.0032) being notably lower compared with the SBM group (Fig. 8A). However, there was no significant difference between the SBM and FM groups in these four indices (*P* = 0.3792, 0.1828, 0.7136, 0.7334). Principal coordinate analysis served to evaluate β-diversity (Fig. 8B), and the Kruskal–Wallis test displayed that notable discrepancies between the three groups of microorganisms (*P* = 0.00968). At the phylum level (Fig. 8C), Fusobacteriota, Pseudomonadota, and Bacillota dominated the intestinal microbiota of juvenile largemouth bass; among them, Fusobacteriota accounted for 68.67% in the YS group, and Pseudomonadota accounted for 54.73% in the SBM group. Dominant genera (Fig. 8D) included *Cetobacterium*, *Plesiomonas*, *Roseateles*, *Acinetobacter*, *Aeromonas*, and *Pseudomonas*. Welch’s *t*-test at genus level (95% confidence interval; Fig. 8E) showed: Significant differences in *Vogesella* abundance between SBM and FM groups. Marked differences in *Edwardsiella*, *Vogesella*, *Paeniglutamicibacter,* and *Steroidobacter* between the SBM and YS groups.

### Metabolomics analysis

**Fig. 9:**
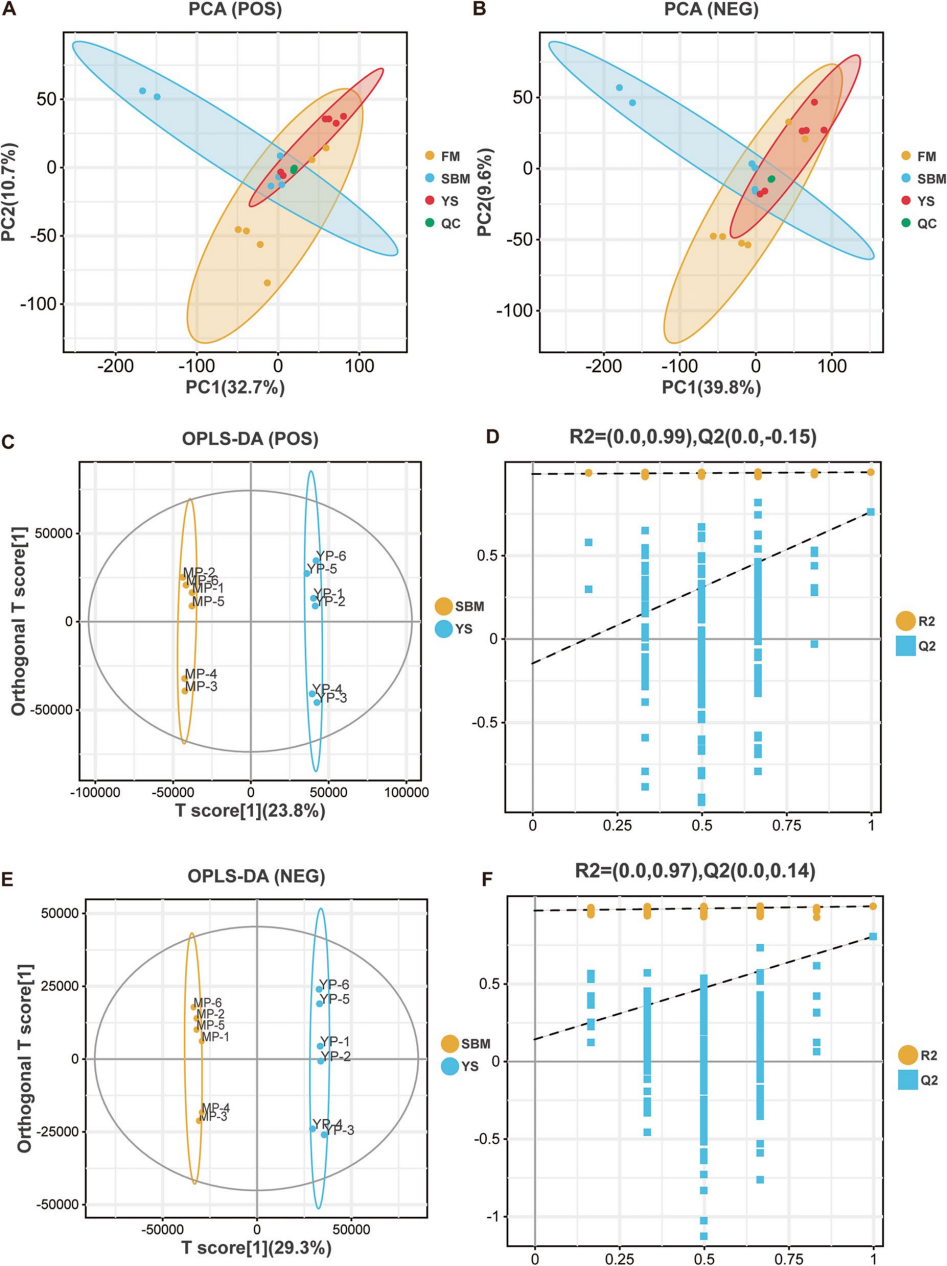
Multivariate statistical analysis in metabolomics (*n* = 6). **A** Principal component analysis for positive ions. **B** Principal component analysis for negative ions. **C** OPLS-DA score plot for positive ions. **D** OPLS-DA permutation test plot for positive ions. **E** OPLS-DA score plot for negative ions. **F** OPLS-DA permutation test plot for negative ions

Based on the comprehensive analysis of growth performance, histological morphology, and physiological and biochemical indicators as described above, the intestinal samples of the FM, SBM, and SBM + 1%YS groups were selected for metabolomic analysis. Multivariate analysis results revealed distinctions in intestinal metabolites across the three groups (Fig. 9A and B), and the tight clustering of quality control samples confirmed the stability of the instrument. OPLS-DA yielded results that could effectively explain and forecast the disparities between the SBM and YS groups (Fig. 9C and E). Specifically, the model validation parameters for positive ion mode were (R^2^X = 0.496, R^2^Y = 0.997, Q^2^ = 0.759), while those for negative ion mode were (R^2^X = 0.615, R^2^Y = 0.997, Q^2^ = 0.718). In addition, we used a permutation test to evaluate the accuracy of the OPLS-DA model (Fig. 9D and F), and the results showed that the model had accurate predictability.

**Fig. 10:**
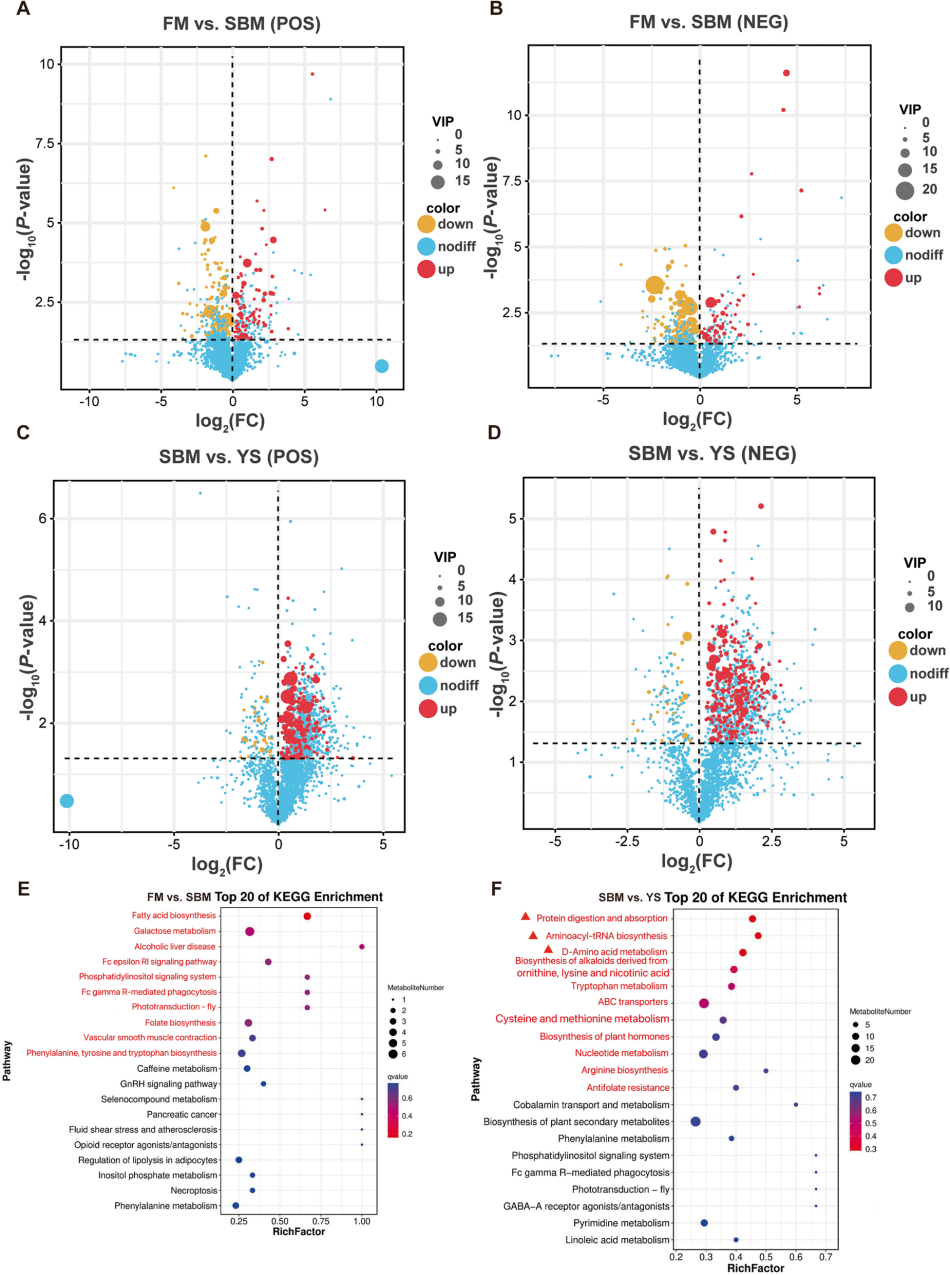
Analysis of differential metabolites and metabolic pathways. **A** Volcano plot of positive ion mode in FM vs. SBM groups. **B** Volcano plot of negative ion mode in FM vs. SBM groups. **C** Volcano plot of positive ion mode in SBM vs. YS groups. **D** Volcano plot of negative ion mode in SBM vs. YS groups. **E** Top 20 of KEGG Enrichment pathways for FM vs. YS. **F** Top 20 of KEGG Enrichment pathways for SBM vs. YS

Based on the metabolomics results, differential metabolites were screened using the dual criteria of VIP value > 1.5 and *P*-value < 0.05. A key finding was the prevalence of downregulated metabolites in the SBM group relative to the FM group (Fig. 10A and B), while after adding YS to the SBM-based diet, a large number of differential metabolites were upregulated (Fig. 10C and D). In positive ion (POS) mode, 200 metabolites exhibited significant upregulation and 20 metabolites showed significant downregulation in the YS group (*P* < 0.001 to *P* = 0.049373). For the negative ion (NEG) mode, the corresponding numbers were 302 significantly upregulated metabolites and 27 significantly downregulated ones in the same group (*P* < 0.001 to *P* = 0.048656). To further explore the differential metabolites and metabolic pathways, an analysis was carried out on the top 20 pathways in terms of enrichment degree in the Kyoto Encyclopedia of Genes and Genomes (KEGG). It was found that there were significant differences in the top 10 pathways in FM vs. SBM group (Fig. 10E), and significant differences in the top 11 pathways in SBM vs. YS group (*P* = 0.004097 to 0.049887) (Fig. 10F). These 11 pathways are specifically as follows: protein digestion and absorption, aminoacyl-tRNA synthesis, D-amino acid metabolism, lysine, and nicotinic acid, biosynthesis of alkaloids derived from ornithine, tryptophan metabolism, ABC transporters, cysteine and methionine metabolism, biosynthesis of plant hormones, nucleotide metabolism, arginine biosynthesis, and folate resistance.

### Correlation analysis between intestinal microbiota and differentially expressed metabolites

**Fig. 11:**
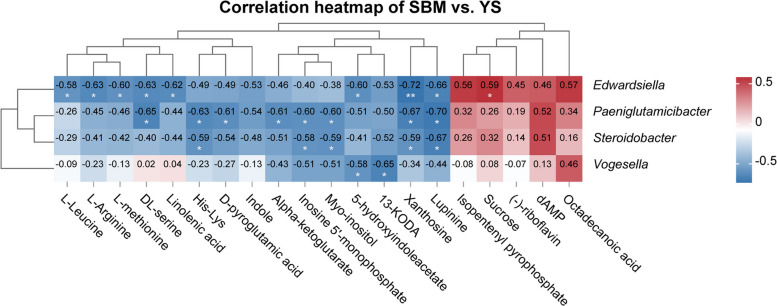
The correlation heatmap of intestinal microbiota and differentially expressed metabolites in the SBM vs. YS groups. Darker red (larger *R* value) and darker blue (lower *R* value) colors indicate stronger positive and negative correlations, respectively. Significant correlations are marked by an asterisk

For a deeper examination of the link between intestinal microbiota and metabolites with differential expression, Pearson correlation analysis was conducted on microbial genera identified by Welch's *t*-test between the SBM and YS groups, and selected differentially expressed metabolites from the top 11 enriched KEGG pathways. The results (Fig. 11) showed that the pathogenic genus *Edwardsiella* was strongly linked to sucrose levels (*R* = 0.59, *P* < 0.05), while it was significantly negatively correlated with amino acids such as leucine and arginine, as well as 5-hydroxyindole, Xanthosine, and lupinine (*R* = −0.72 to −0.58, *P* < 0.05). The conditional pathogenic genera *Paeniglutamicibacter* and *Steroidobacter* showed highly similar correlation patterns, while *Vogesella* was negatively correlated with 5-hydroxyindole and 13-keto-9Z,11E-octadecadienoic acid (13-KODA) (*R* = −0.58 to −0.65, *P* < 0.05).

### Correlation analysis of growth performance and immune indicators with multi-omics indicators

**Fig. 12:**
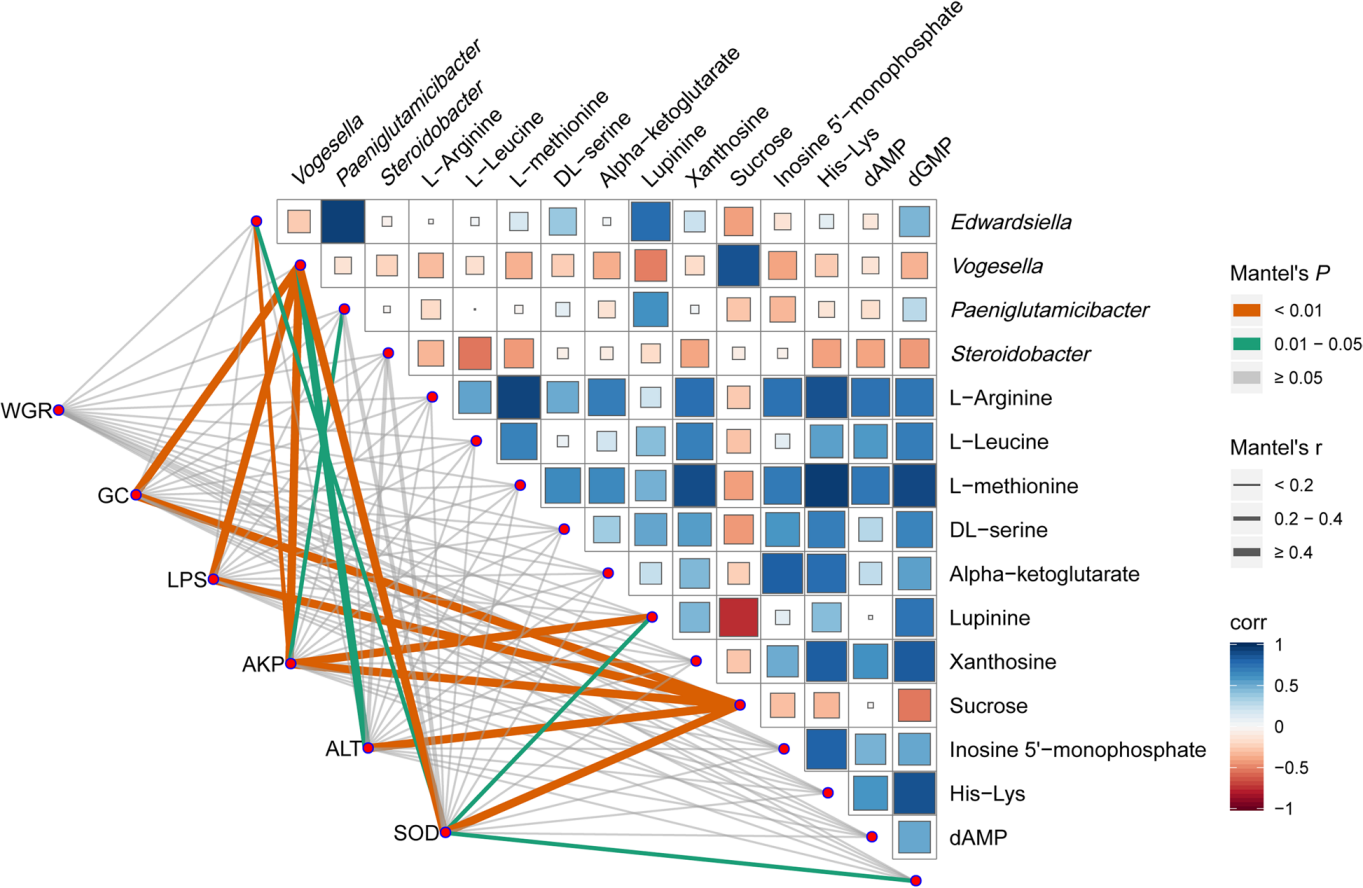
Relationships between growth performance, immune indices, differential intestinal flora, and differential metabolites in juvenile largemouth bass revealed by the Mantel test. Edge width corresponds to Mantel's *r* value, and the edge color denotes statistical significance. Pairwise correlations of these variables are shown with a color gradient denoting Spearman's correlation coefficient. WGR, weight gain rate; GC, goblet cell; LPS, lipopolysaccharide; AKP, alkaline phosphatase; ALT, alanine transaminase; SOD, superoxide dismutase

The Mantel test was used to explore the associations between factors such as growth performance, immunity, gut microbiota, and differential metabolites (Fig. 12). Among them, GC, LPS, AKP, and SOD were significantly correlated with *Vogesella* (*r* = 0.43 to 0.69, *P* = 0.002, 0.003, 0.01, 0.009). Similarly, GC, LPS, AKP, ALT, and SOD were significantly correlated with Sucrose (*r* = 0.49 to 0.69, *P* = 0.003, 0.002, 0.001, 0.01, 0.007).

## Discussion

### Effects of yeast enzyme hydrolysis slurry on growth performance and digestive ability of juvenile largemouth bass

Current studies demonstrate that dietary supplementation of 1%–2% yeast enzyme hydrolysis slurry in soybean meal-based diets with partial fishmeal replacement promotes growth performance in juvenile largemouth bass. Yeast-derived additives, such as yeast hydrolysate, yeast culture, and yeast cell wall, have all been shown to enhance the growth performance of aquatic animals [14, 25, 26]. In this study, the efficacy of the 1% YS-supplemented diet was comparable to that of the FM group. This further validates the universal application of yeast-derived products in low-fishmeal diets for aquatic species [27]. The growth-promoting effect of YS is presumably closely associated with its unique nutritional composition: processed via enzymatic hydrolysis, this product is rich in functional components that are easily digested and absorbed by aquatic animals, such as free amino acids, nucleotides, and small-molecule peptides [28–30].

The capacity of fish to break down and absorb nutrients is directly reflected by intestinal digestive enzymes [31]. In this study, YS supplementation in soybean meal-based diets with partial fishmeal replacement significantly enhanced digestive enzyme activities. YS may exert growth-promoting effects via its abundant bioactive components. Previous studies have demonstrated that yeast-derived products typically contain functional substances such as amino acids, oligosaccharides, and nucleotides [32], which have been proven to enhance intestinal health and digestive function in fish. For instance, dietary supplementation of yeast β-glucan can significantly increase the intestinal fold height, muscle layer thickness, and digestive enzyme activity in coho salmon (*Oncorhynchus kisutch*) [33].

Appetite and feed intake in teleosts are regulated by interactions between orexigenic factors and anorexigenic factors [34]. Research indicates that downregulation of hypothalamic anorexigenic factors can enhance appetite and increase the food consumption of different types of fish [35]. This research revealed that the expression levels of anorexigenic genes *cck* and *lep* in the SBM group were reduced by YS supplementation. This significant downregulation is the core mechanism underlying YS-induced improvements in appetite and feeding performance. This effect is presumably closely associated with the rich amino acid composition of YS: glutamic acid, a flavor-enhancing amino acid [36], can act synergistically with aspartic acid as an excitatory neurotransmitter to directly modulate appetite signaling by targeting the hypothalamic region of the brain. Meanwhile, its degradation metabolites–glutamine, γ-aminobutyric acid (GABA), and α-ketoglutarate–may further potentiate the inhibition of anorexigenic pathways by regulating leptin secretion [37]. Although previous studies have shown that leptin can suppress the mRNA expression of the orexigenic gene *npy* [38], and an inverse expression trend between *npy* and *lep* was observed in this study, the changes in *npy* expression did not reach statistical significance. Thus, *npy* might not be the primary driver of YS-mediated feeding regulation.

In summary, YS enhances appetite and feed intake in juvenile largemouth bass primarily by significantly downregulating the expression of *cck* and *lep* thereby inhibiting anorexigenic pathways. However, further research is needed to elucidate whether YS-mediated regulation of anorexigenic factors involves other signaling pathways, such as metabolic signaling transduction via the gut-brain axis.

### Effects of yeast enzyme hydrolysis slurry on intestinal histomorphology and antioxidant capacity in juvenile largemouth bass

The morphological structure of the intestine is essential for the absorption of nutrients and sustaining intestinal functionality [39]. Generally, intestinal fold height and depth reflect the nutrient absorption efficiency of the digestive system [40]. Intestinal fold height increases contact surface area with nutrients, muscularis thickness indicates intestinal contraction strength, and goblet cells play key roles in maintaining epithelial integrity and regulating immune responses to exogenous antigens [41]. In this study, compared to the soybean meal group, YS supplementation significantly increased intestinal fold height, muscularis thickness, and goblet cell numbers in the foregut, midgut, and hindgut. Similar studies have shown that dietary supplementation of 1% yeast culture significantly increased the intestinal fold width in the foregut and hindgut, as well as the muscularis thickness in the midgut and hindgut of *Cyprinus carpio* [42].

The DAO, D-Lac, and LPS are biomarkers of damage to the intestinal mucosal layer [43]. In this study, dietary YS significantly reduced DAO, D-Lac, and LPS levels, thereby decreasing intestinal permeability in largemouth bass. Intestinal barrier permeability directly reflects gut health, primarily determined by tight junction integrity [44]. Tight junction damage often accompanies increased oxidative stress [45], leading to ROS accumulation. Studies indicate ROS accumulation downregulates antioxidant enzymes (CAT, GSH-Px, and SOD) and elevates MDA in fish tissues [46]. For example, Dietary yeast extracts enhanced CAT and SOD activity in Chinese mitten crab (*Eriocheir sinensis*) [47]. Yeast products increased plasma SOD activity in *Aeromonas hydrophila*-challenged silver carp (*Hypophthalmichthys molitrix*) [48]. In addition, lysozyme activity in head kidney macrophages of Atlantic salmon (*Salmo salar*) was elevated by yeast β-glucan [49]. Dietary nucleotides improved lysozyme and SOD activity in whiteleg shrimp (*Litopenaeus vannamei*) [50]. Thus, yeast enzyme hydrolysis slurry can ameliorate intestinal histomorphology and boost antioxidant capacity in juvenile largemouth bass. However, this study did not involve research on organelles in intestinal tissue sections.

### Effects of yeast enzyme hydrolysis slurry on intestinal microbiota in juvenile largemouth bass

In the regulation of host metabolism, immunity, and intestinal health, the intestinal microbiota plays an essential role [51]. Notably, dietary YS supplementation did not significantly increase microbial diversity and richness but instead showed a declining trend. At the phylum level, Fusobacteriota, Pseudomonadota, and Bacillota dominated the microbiota; this aligns with prior research on largemouth bass [52]. The YS group exhibited significantly increased beneficial Fusobacteriota and decreased harmful Pseudomonadota, explaining the diversity shift. At the genus level, YS enhanced beneficial *Cetobacterium* while suppressing the abundance of pathogenic bacteria, including *Plesiomonas, Acinetobacter, *and* Pseudomonas* [53–55]. *Cetobacterium* aids digestion, vitamin B_12_ synthesis, and inhibits pathogenic colonization [56, 57]. Additionally, Welch's *t*-test conducted at the genus level revealed that the SBM group had notably higher *Vogesella* abundance than the FM and YS groups, and notably higher abundance of *Edwardsiella*, *Paeniglutamicibacter*, and *Steroidobacter* than the YS group. *Vogesella* is a common genus closely associated with the white discoloration syndrome in crustacean gastrointestinal tracts, and its significant increase in the SBM group reflects dysbiosis [58]. *Edwardsiella*, a pathogenic bacterium of black sea bass [59], showed a significant decrease in abundance after YS addition. A plausible explanation is that *Cetobacterium* enriched in the YS group produces antimicrobial peptides and lowers intestinal pH, thereby reducing the colonization and proliferation of potential pathogenic bacteria [60, 61]. The dominant putrefactive bacterium genus *Paeniglutamicibacter* and the dominant bacterium genus *Steroidobacter* during meat freezing processes were also significantly enriched in the SBM group [62, 63]. In summary, dietary YS can improve the health of largemouth bass intestines by significantly elevating the abundance level of beneficial bacterial communities and lowering that of harmful bacterial communities. However, the YS group did not directly lead to a significant increase in the abundance and diversity of intestinal microbiota. This differs from conventional yeast additives that typically enhance microbial abundance [14, 42]. A plausible explanation is that the unique processing technology of YS facilitates the enrichment of beneficial bacteria and inhibits the colonization of potential pathogenic bacteria [64].

### Effects of yeast enzyme hydrolysis slurry on metabolomics in juvenile largemouth bass

In this study, PCA was applied to examine the distribution pattern of intestinal metabolites among different groups. From the PCA results, it could be observed that intestinal metabolite distribution in the YS group shared a high degree of overlap with the FM group, while differing from what was seen in the SBM group. The volcano plot showed the upregulation and downregulation of differential metabolites between the SBM and YS groups after screening.

In the protein digestion and absorption pathway, differential metabolites such as L-arginine, L-tyrosine, L-leucine, and L-methionine were significantly upregulated. Among them, L-arginine, an essential amino acid for fish, and L-leucine can promote protein synthesis and improve growth performance by activating the mTOR signaling pathway [65]. Yeast extract may enhance the adaptability of largemouth bass to plant proteins by improving the bioavailability of arginine in plant protein feeds [66]. Arginine is also the sole substrate for nitric oxide (NO) production in the body, assisting in the generation of NO to scavenge reactive oxygen species (ROS) and enhance the antioxidant capacity of fish [67]. L-Methionine has been shown to alleviate the negative effects of reduced antioxidant capacity in largemouth bass fed low-fishmeal diets [68]. Additionally, some undigested and unabsorbed peptides in this metabolic pathway can produce differentially expressed metabolites such as amines and indoles through amino acid degradation and fatty acid production, which are significantly upregulated to promote digestion, absorption, and intestinal health. Protein biosynthesis is a key process in various vital activities, and aminoacyl-tRNA synthetases play a crucial role [69]. In the D-amino acid metabolism pathway, differentially expressed metabolites such as 2-oxoglutarate, D-serine, D-glutamine, and L-threonine were significantly upregulated. 2-Oxoglutarate, an intermediate of the tricarboxylic acid cycle, is involved in energy metabolism and amino acid synthesis. D-Glutamine can promote the proliferation of intestinal cells, maintain the expression level of tight junction proteins, repair the mucosal barrier, reduce intestinal inflammation, and enhance the tolerance of fish to soybean meal-based diets [70, 71]. D-Serine and L-threonine jointly participate in the metabolism of glycine, serine, and threonine, which is related to microbial growth and involved in the inhibition of pathogens [72]. Taken together, yeast enzyme hydrolysis slurry can improve the growth and intestinal health of juvenile largemouth bass through the regulation of the upregulation and downregulation of differentially expressed metabolites.

### Correlation analysis of intestinal microbiota and intestinal metabolomics

The intestinal microbiota affects digestion and absorption, which in turn alters the metabolic activities of the organism [73]. Through the analysis of variations in intestinal flora and metabolites, we can better explore the effects of enzyme hydrolysis of yeast pulp on the growth, metabolism, and intestinal health of largemouth bass. Between the metabolite sucrose and the pathogen *Edwardsiella* in largemouth bass, a positive correlation was identified through correlation analysis. Studies have shown that sucrose itself does not directly regulate the virulence genes of *Edwardsiella*, but may jointly affect the pathogenicity of *Edwardsiella* with the metabolism of other carbon sources in the intestine. For example, glycolipid metabolic disorders caused by high-starch diets may indirectly weaken host immunity and increase the risk of *Edwardsiella* infection [74]. *Edwardsiella* may primarily depend on carbohydrate metabolites to carry out infection, whereas the host might activate amino acid metabolic pathways as a mean of self-defense [75], so amino acids in the heat map showed a negative correlation with *Edwardsiella*, which also explains why amino acid metabolic pathways were significantly enriched after the addition of enzyme hydrolysis of yeast pulp. However, *Paeniglutamicibacter* and *Steroidobacter* have not been deeply studied in largemouth bass, and only a few studies have shown that both are dominant spoilage bacteria [62, 63]. The significant negative correlation between the *Vogesella* genus and 5-hydroxyindole, 13-keto-9Z,11E-octadecadienoic acid suggests its disruptive effect on intestinal barrier and immune homeostasis. 5-Hydroxyindole is an important metabolite of tryptophan, which promotes intestinal barrier repair by activating the aryl hydrocarbon receptor (AhR) [76]. The 13-KODA, as a derivative of linoleic acid, participates in regulating intestinal inflammatory responses and epithelial cell integrity [77]. The significant correlation between the *Vogesella* genus and GC, SOD, and other immune factors in the Mantel test further demonstrates that the *Vogesella* genus may be a key group triggering intestinal microbiota dysbiosis and immune suppression in largemouth bass.

Additionally, the Mantel test showed a significant correlation between GC, LPS, AKP, SOD, and sucrose, indicating that the negative effects of sucrose on host physiology are not limited to serving as a carbon source for pathogenic bacteria but may also directly interfere with the immune function of juvenile largemouth bass. For example, a high-sucrose environment may induce oxidative stress in intestinal epithelial cells, reducing the antioxidant activity of SOD, while sucrose metabolic disorders may disrupt intestinal mucosal structure [78]. Therefore, YS can effectively alleviate intestinal barrier damage caused by plant protein feed by reducing sucrose levels and the abundance of harmful bacteria. However, the correlation analysis lacks the association with genes.

## Conclusion

In this study, the inclusion of yeast enzyme hydrolysis slurry in soybean meal-based diets with partial fishmeal replacement enhanced the digestion and antioxidant capacity, reduced intestinal permeability, altered the abundance of intestinal microbiota and associated core metabolites. These positive changes collectively contributed to improved growth performance in largemouth bass. Correlation analysis further indicated that differential metabolites such as arginine and methionine were significantly negatively correlated with *Edwardsiella*, suggesting a potential mechanism by which YS mitigates the adverse effects of high soybean inclusion. Based on our findings, we recommend supplementing 1% YS in soybean meal-based diets with reduced fishmeal as an effective strategy to enhance growth and intestinal health in largemouth bass. To further validate the practical application of YS and elucidate its underlying mechanisms, future studies should: (i) conduct a full-scale growth trial to evaluate the long-term effects of YS on growth performance, feed utilization, and overall health under practical farming conditions; (ii) perform targeted challenge tests to verify the role of YS in improving disease resistance; and (iii) integrate metabolomic and microbiome analyses to clarify the key pathways through which YS modulates host metabolism and microbiota interactions. This study provides a new perspective for understanding the host-microbiota-metabolite interactions in high-plant-protein diets and offers theoretical and practical support for developing fishmeal replacement strategies to advance sustainable aquaculture of largemouth bass.

## Data Availability

All data generated or analyzed during this study are available from the corresponding author upon reasonable request.

## Abbreviations

ACP: Acid phosphatase
AKP: Alkaline phosphatase
ALT: Alanine transaminase
AOAC: Association of Official Analytical Chemists
AST: Aspartate transaminase
CAT: Catalase
*cck*: Cholecystokinin
CF: Condition factor
DAO: Diamine oxidase
D-Lac: D-Lactate
ELISA: Enzyme-linked immunosorbent assay
FCR: Feed conversion ratio
FM: Fish meal
FR: Feeding ratio
GABA: Glutamine, γ-aminobutyric acid
GC: Goblet cell
GSH-Px: Glutathione peroxidase
HIS: Hepatosomatic index
ISI: Intestine somatic index
KEGG: Kyoto Encyclopedia of Genes and Genomes
*lep*: Leptin
LPS: Lipopolysaccharide
MLT: Muscular layer thickness
mTOR: Mammalian target of rapamycin
NEG: Negative ion mode
*npy*: Neuropeptide Y
NO: Nitric oxide
OPLS-DA: Orthogonal partial least squares discriminant analysis
PCoA: Principal coordinates analysis
PER: Protein efficiency rate
IFH: Intestinal fold height
POS: Positive ion mode
IFW: Intestinal fold width
ROS: Reactive oxygen species
SBM: Soybean meal
SGR: Specific growth rate
SOD: Superoxide dismutase
T-AOC: Total antioxidant capacity
VIP: Variable importance in projection
VSI: Viscera somatic index
WGR: Weight gain rate
YS: Yeast enzyme hydrolysis slurry
13-KODA: 13-Keto-9Z,11E-octadecadienoic acid

## References

[1] Cheng Y, Wu YJ, Zhu CB, Zhou XK, Yang S, Fei H. Alternative proteins as fish meal substitution in diets for largemouth bass (*Micropterus salmoides*): a mini review. Int J Food Sci Technol. 2023;58:5449–58. 10.1111/ijfs.16471.

[2] Hussain SM, Bano AA, Ali S, Rizwan M, Adrees M, Zahoor AF, et al. Substitution of fishmeal: Highlights of potential plant protein sources for aquaculture sustainability. Heliyon. 2024;10:26573. 10.1016/j.heliyon.2024.e26573. 10.1016/j.heliyon.2024.e26573 PMC10906437 38434023

[3] Ali A, Deverajan S, Manickavasagan A, Ata A. Antinutritional factors and biological constraints in the utilization of plant protein foods. In: Manickavasagan A, Lim LT, Ali A, editors. Plant protein foods. Cham: Springer; 2022. p. 407–38. 10.1007/978-3-030-91206-2_14.

[4] Francis G, Makkar HPS, Becker K. Antinutritional factors present in plant-derived alternate fish feed ingredients and their effects in fish. Aquaculture. 2001;199:197–227. 10.1016/S0044-8486(01)00526-9.

[5] Coutinho F, Castro C, Rufino-Palomares E, Ordóñez-Grande B, Gallardo MA, Oliva-Teles A, et al. Dietary glutamine supplementation effects on amino acid metabolism, intestinal nutrient absorption capacity and antioxidant response of gilthead sea bream (*Sparus aurata*) juveniles. Comp Biochem Physiol A Mol Integr Physiol. 2016;191:9–17. 10.1016/j.cbpa.2015.09.012. 26424608 10.1016/j.cbpa.2015.09.012

[6] Li X, Wei X, Guo X, Mi S, Hua X, Li N, et al. Enhanced growth performance, muscle quality and liver health of largemouth bass (*Micropterus salmoides*) were related to dietary small peptides supplementation. Aquacult Nutr. 2020;26:2169–77. 10.1111/anu.13155.

[7] Liu L, Fang J, Liang X, He S. Nucleotide promotes feed intake and protein utilization via regulating the gene expression of feeding and nitrogen metabolism in juvenile Chinese perch (*Siniperca chuatsi*). Aquacult Nutr. 2020;26:1702–12. 10.1111/anu.13121.

[8] Fu L, Han D, Yi J, Zhang Z, Liu H, Jin J, et al. Effects of dietary yeast hydrolysate on the growth performance, intestine health and digestion of juvenile yellow catfish (*Pelteobagrus vachelli ♂* × *Pelteobagrus fulvidraco* ♀). Aquacult Rep. 2023;29:101496. 10.1016/j.aqrep.2023.101496

[9] Gong Y, Yang F, Hu J, Liu C, Liu H, Han D, et al. Effects of dietary yeast hydrolysate on the growth, antioxidant response, immune response and disease resistance of largemouth bass (*Micropterus salmoides*). Fish Shellfish Immunol. 2019;94:548–57. 10.1016/j.fsi.2019.09.044. 31539573 10.1016/j.fsi.2019.09.044

[10] Rahimnejad S, Leclercq E, Malinovskyi O, Pěnka T, Kolářová J, Policar T. Effects of yeast hydrolysate supplementation in low-fish meal diets for pikeperch. Animal. 2023;17:100870. 10.1016/j.animal.2023.100870. 37379608 10.1016/j.animal.2023.100870

[11] Frohn L, Peixoto D, Guyomar C, Teixeira C, Terrier F, Aguirre P, et al. Yeast extract improves growth in rainbow trout (*Oncorhynchus mykiss*) fed a fishmeal-free diet and modulates the hepatic and distal intestine transcriptomic profile. Aquaculture. 2024;579:740226. 10.1016/j.aquaculture.2023.740226.

[12] Wang T, Yang J, Lin G, Li M, Zhu R, Yiannikouris A, et al. Evaluation of the mitigation efficacy of a yeast cell wall extract toward deoxynivalenol contaminated diet fed to turbot (*Scophthalmus** maximus*). Ecotoxicol Environ Saf. 2021;216:112221. 10.1016/j.ecoenv.2021.112221. 33862437 10.1016/j.ecoenv.2021.112221

[13] Yu Z, Zhang Z, Teame T, Guan L, Wang R, Zhu R, et al. Yeast cell wall extract as a strategy to mitigate the effects of aflatoxin B1 and deoxynivalenol on liver and intestinal health, and gut microbiota of largemouth bass (*Micropterus salmoides*). Aquaculture. 2025;597:741917. 10.1016/j.aquaculture.2024.741917.

[14] Wang S, Li E, Yu Q, Luo Z, Li W, Wang X, et al. Supplementation of yeast culture to low-fishmeal diets improves growth, intestinal health, and heat stress resistance in juvenile Chinese mitten crab (*Eriocheir sinensis*). Aquaculture. 2024;593:741308. 10.1016/j.aquaculture.2024.741308.

[15] Duan Z, Chen Y, Li X, Cao K, Zhao Y, Yang X, et al. An evaluation of yeast culture in diet of hybrid snakehead (*Channa argus ♂ × Channa maculate ♀*): growth, serum biochemical parameters, intestinal histology and microorganisms. Aquac Rep. 2023;32:101711. 10.1016/j.aqrep.2023.101711.

[16] Thakkar A, Barbera E, Sforza E, Bertucco A, Davis R, Kumar S. Flash hydrolysis of yeast (*Saccharomyces cerevisiae*) for protein recovery. J Supercrit Fluids. 2021;173:105240. 10.1016/j.supflu.2021.105240.

[17] Xie J, Cui C, Ren J, Zhao M, Zhao L, Wang W. High solid concentrations facilitate enzymatic hydrolysis of yeast cells. Food Bioprod Process. 2017;103:114–21. 10.1016/j.fbp.2017.03.004.

[18] Ye Y. Reforming quality of feed raw materials with new technologies, and reforming quality and technology of aquatic feed with new raw materials. FEED Ind. 2025;46:1–10. 10.13302/j.cnki.fi.2025.08.001. (In Chinese).

[19] Bureau of Fisheries, Ministry of Agriculture and Rural Affairs of P. R. China, National Fisheries Technology Extension Center, China Society of Fisheries. China Fishery Statistical Yearbook. Beijing: China Agriculture Press; 2025.

[20] Bai J, Li S. Development of largemouth bass (*Micropterus salmoides*) culture. In: Gui JF, Tang QS, Li ZJ, Liu JS, De Silva SS, editors. Aquaculture in China: Success stories and modern trends. Wiley & Sons Ltd. 2018. p. 421–9. 10.1002/9781119120759.ch4_5.

[21] Feng Z, Zhong Y, He G, Sun H, Chen Y, Zhou W, et al. Yeast culture improved the growth performance, liver function, intestinal barrier and microbiota of juvenile largemouth bass (*Micropterus salmoides*) fed high-starch diet. Fish Shellfish Immunol. 2022;120:706–15. 10.1016/j.fsi.2021.12.034. 34954371 10.1016/j.fsi.2021.12.034

[22] Wang Y, Wang J, Liu L, Xu H, Liang H, Wang Z, et al. Effects of dietary yeast nucleotides on the growth performance and muscle quality of juvenile largemouth bass (*Micropterus salmoides*). Aquac Rep. 2024;36:102159. 10.1016/j.aqrep.2024.102159.

[23] AOAC. Official Methods of Analysis. 18th ed. Gaithersburg: AOAC International; 2005.

[24] Livak KJ, Schmittgen TD. Analysis of relative gene expression data using real-time quantitative PCR and the 2−ΔΔCT method. Methods San Diego Calif. 2001;25:402–8. 10.1006/meth.2001.1262. 11846609 10.1006/meth.2001.1262

[25] Kong Y, Gao Q, Zhou D, Feng Q, Ding Z, Limbu SM, et al. Dietary yeast hydrolysate improves growth performance, antioxidant capacity, immunity, and intestinal microbiota of juvenile giant freshwater prawn (*Macrobrachium rosenbergii*). Anim Feed Sci Technol. 2025;319:116196. 10.1016/j.anifeedsci.2024.116196.

[26] Huang W, Xiao X, Hu W, Tang T, Bai J, Zhao S, et al. Effects of dietary nucleotide and yeast cell wall on growth performance, feed utilization, anti-oxidative and immune response of grass carp (*Ctenopharyngodon idella*). Fish Shellfish Immunol. 2023;134:108574. 10.1016/j.fsi.2023.108574. 36731810 10.1016/j.fsi.2023.108574

[27] Türker M, Derman ÜC, Alemdar F. 17 - Yeast-derived products and their commercial applications. In: González-Fernández C, Tomás-Pejó E, editors. Eukaryotic Microorganisms as Sources of Bioproducts (Second Edition). Woodhead Publishing; 2025. p. 455–78. 10.1016/B978-0-443-30188-9.00007-X.

[28] Hossain MdS, Koshio S, Ishikawa M, Yokoyama S, Sony NM. Dietary nucleotide administration influences growth, immune responses and oxidative stress resistance of juvenile red sea bream (*Pagrus major*). Aquaculture. 2016;455:41–9. 10.1016/j.aquaculture.2016.01.008.

[29] Rumsey GL, Winfree RA, Hughes SG. Nutritional value of dietary nucleic acids and purine bases to rainbow trout (*Oncorhynchus mykiss*). Aquaculture. 1992;108:97–110. 10.1016/0044-8486(92)90321-B.

[30] Suresh AV, Nates S. Attractability and palatability of protein ingredients of aquatic and terrestrial animal origin, and their practical value for blue shrimp, *Litopenaeus stylirostris* fed diets formulated with high levels of poultry byproduct meal. Aquaculture. 2011;319:132–40. 10.1016/j.aquaculture.2011.06.039.

[31] Fu W, Amenyogbe E, Luo J, Yang E, Huang J, Chen Y, et al. Influences of ferulic acid on intestinal digestive and antioxidant enzymes, immune, antioxidant gene and tight junction protein expression and microbiota in hybrid grouper (*Epinephelus fuscoguttatus*♀× *Epinephelus polyphekadion*♂). Aquac Rep. 2022;27:101348. 10.1016/j.aqrep.2022.101348.

[32] Perricone V, Sandrini S, Irshad N, Savoini G, Comi M, Agazzi A. Yeast-derived products: the role of hydrolyzed yeast and yeast culture in poultry nutrition—a review. Animals. 2022;12:1426. 10.3390/ani12111426. 35681890 10.3390/ani12111426 PMC9179594

[33] Shi Y, Kong W, Gong F, Cai C, Zhang Y, Cheng G, et al. Yeast β-glucan enhances the intestinal immune function in coho salmon *via* the modulation of gut microbiota-mediated lipid metabolism. Aquaculture. 2025;599:742123. 10.1016/j.aquaculture.2025.742123.

[34] Rønnestad I, Gomes AS, Murashita K, Angotzi R, Jönsson E, Volkoff H. Appetite-controlling endocrine systems in teleosts. Front Endocrinol. 2017;8:73. 10.3389/fendo.2017.00073. 10.3389/fendo.2017.00073 PMC5394176 28458653

[35] Volkoff H, Canosa LF, Unniappan S, Cerdá-Reverter JM, Bernier NJ, Kelly SP, et al. Neuropeptides and the control of food intake in fish. Gen Comp Endocrinol. 2005;142:3–19. 10.1016/j.ygcen.2004.11.001. 15862543 10.1016/j.ygcen.2004.11.001

[36] Morais S. The physiology of taste in fish: potential implications for feeding stimulation and gut chemical sensing. Rev Fish Sci Aquac. 2017;25:133–49. 10.1080/23308249.2016.1249279.

[37] Romaní-Pérez M, Bullich-Vilarrubias C, López-Almela I, Liébana-García R, Olivares M, Sanz Y. The microbiota and the Gut–Brain axis in controlling food intake and energy homeostasis. Int J Mol Sci. 2021;22:5830. 10.3390/ijms22115830. 10.3390/ijms22115830 PMC8198395 34072450

[38] Schwartz M, Seeley R, Woods S, Weigle D, Campfield L, Burn P, et al. Leptin increases hypothalamic pro-opiomelanocortin mRNA expression in the rostral arcuate nucleus. Diabetes. 1997;46:2119–23. 10.2337/diab.46.12.2119. 9392508 10.2337/diab.46.12.2119

[39] Fang H, Xie J, Liao S, Guo T, Xie S, Liu Y, et al. Effects of dietary inclusion of shrimp paste on growth performance, digestive enzymes activities, antioxidant and immunological status and intestinal morphology of hybrid snakehead (*Channa maculata* ♀ × *Channa argus* ♂). Front Physiol. 2019. 10.3389/fphys.2019.01027. 10.3389/fphys.2019.01027 PMC6693359 31440171

[40] Zhang J, Wang Z, Shi Y, Xia L, Hu Y, Zhong L. Protective effects of chlorogenic acid on growth, intestinal inflammation, hepatic antioxidant capacity, muscle development and skin color in channel catfish *Ictalurus punctatus* fed an oxidized fish oil diet. Fish Shellfish Immunol. 2023;134:108511. 10.1016/j.fsi.2022.108511. 36599381 10.1016/j.fsi.2022.108511

[41] Yang S, Yu M. Role of goblet cells in intestinal barrier and mucosal immunity. J Inflamm Res. 2021;14:3171–83. 10.2147/JIR.S318327. 34285541 10.2147/JIR.S318327 PMC8286120

[42] Tao S, Wang J, Xue Z, Bai Y, Wang Y, Zhou W, et al. The improved growth performance of *Cyprinus carpio* by dietary yeast culture depends on improvement of intestinal structure and digestive enzyme activities rather than changes of intestinal microbiota composition. Aquac Rep. 2025;44:103053. 10.1016/j.aqrep.2025.103053.

[43] Li C, Li X, Li P, Wei B, Zhang C, Zhu X, et al. Sodium humate alters the intestinal microbiome, short-chain fatty acids, eggshell ultrastructure, and egg performance of old laying hens. Front Vet Sci. 2022. 10.3389/fvets.2022.986562. 10.3389/fvets.2022.986562 PMC9597201 36311664

[44] Ding Q, Hao Q, Jin Y, Zhang Q, Xie Y, Yang Y, et al. The effects of sodium propionate on intestinal barrier function of genetically improved farmed tilapia in a high-lipid formulation. Aquaculture. 2024;579:740187. 10.1016/j.aquaculture.2023.740187.

[45] Yin H, Li R, Liu J, Sun Y, Zhao L, Mou J, et al. Fucosylated chondroitin sulfate from sea cucumber *Stichopus chloronotus* alleviate the intestinal barrier injury and oxidative stress damage *in vitro* and *in vivo*. Carbohydr Polym. 2024;328:121722. 10.1016/j.carbpol.2023.121722. 38220325 10.1016/j.carbpol.2023.121722

[46] Lee JW, Choi H, Hwang UK, Kang JC, Kang YJ, Kim KI, et al. Toxic effects of lead exposure on bioaccumulation, oxidative stress, neurotoxicity, and immune responses in fish: a review. Environ Toxicol Pharmacol. 2019;68:101–8. 10.1016/j.etap.2019.03.010. 30884452 10.1016/j.etap.2019.03.010

[47] Zhang R, Jiang Y, Zhou L, Chen Y, Wen C, Liu W, et al. Effects of dietary yeast extract supplementation on growth, body composition, non-specific immunity, and antioxidant status of Chinese mitten crab (*Eriocheir sinensis*). Fish Shellfish Immunol. 2019;86:1019–25. 10.1016/j.fsi.2018.12.052. 30590164 10.1016/j.fsi.2018.12.052

[48] Zhang P, Cao S, Zou T, Han D, Liu H, Jin J, et al. Effects of dietary yeast culture on growth performance, immune response and disease resistance of gibel carp (*Carassius auratus gibelio* CAS Ⅲ). Fish Shellfish Immunol. 2018;82:400–7. 10.1016/j.fsi.2018.08.044. 30144566 10.1016/j.fsi.2018.08.044

[49] Paulsen SM, Engstad RE, Robertsen B. Enhanced lysozyme production in Atlantic salmon (*Salmo salar* L.) macrophages treated with yeast β-glucan and bacterial lipopolysaccharide. Fish Shellfish Immunol. 2001;11:23–37. 10.1006/fsim.2000.0291. 11271600 10.1006/fsim.2000.0291

[50] Guo J, Guo B, Zhang H, Xu W, Zhang W, Mai K. Effects of nucleotides on growth performance, immune response, disease resistance and intestinal morphology in shrimp *Litopenaeus vannamei* fed with a low fish meal diet. Aquacult Int. 2016;24:1007–23. 10.1007/s10499-015-9967-7.

[51] Diwan AD, Harke SN, Panche AN. Host-microbiome interaction in fish and shellfish: an overview. Fish Shellfish Immunol Rep. 2023;4:100091. 10.1016/j.fsirep.2023.100091. 37091066 10.1016/j.fsirep.2023.100091 PMC10113762

[52] Zhou M, Liang R, Mo J, Yang S, Gu N, Wu Z, et al. Effects of brewer’s yeast hydrolysate on the growth performance and the intestinal bacterial diversity of largemouth bass (*Micropterus salmoides*). Aquaculture. 2018;484:139–44. 10.1016/j.aquaculture.2017.11.006.

[53] Cao B, Yan J, Santos JA. Chapter 50 - Plesiomonas. In: Tang YW, Hindiyeh MY, Liu D, Sails A, Spearman P, Zhang JR, editors. Molecular medical microbiology. Third Ed. Academic Press; 2024. p. 1027–42. 10.1016/B978-0-12-818619-0.00025-3.

[54] Duman M, Lalucat J, Burcin Saticioglu I, Mulet M, Gomila M, Altun S, et al. Description of three new *Pseudomonas* species isolated from aquarium fish: *Pseudomonas auratipiscis* sp. nov., *Pseudomonas carassii* sp. nov. and *Pseudomonas ulcerans* sp. nov. Syst Appl Microbiol. 2024;47:126552. 10.1016/j.syapm.2024.126552. 39340979 10.1016/j.syapm.2024.126552

[55] Walczak N, Puk K, Guz L. Bacterial flora associated with diseased freshwater ornamental fish. J Vet Res. 2017;61:445–9. 10.1515/jvetres-2017-0070. 29978108 10.1515/jvetres-2017-0070 PMC5937343

[56] Larsen AM, Mohammed HH, Arias CR. Characterization of the gut microbiota of three commercially valuable warmwater fish species. J Appl Microbiol. 2014;116:1396–404. 10.1111/jam.12475. 24529218 10.1111/jam.12475

[57] Zhu CB, Shen YT, Ren CH, Yang S, Fei H. A novel formula of herbal extracts regulates growth performance, antioxidant capacity, intestinal microbiota and resistance against *Aeromonas veronii* in largemouth bass (*Micropterus salmoides*). Aquaculture. 2024;583:740614. 10.1016/j.aquaculture.2024.740614.

[58] Cai M, Shao C, He Z, Chang R, Zhang H, Hu Y. Soybean meal-refined treatment mitigated high soybean meal diet-induced oxidative damage in the gut of crayfish via microbial metabolic function remodeling. Aquaculture. 2025;601:742286. 10.1016/j.aquaculture.2025.742286.

[59] Lin C, Jiang B, Huang W, Wu W, Zhang J, Su Y. Environmental adaptability, virulence, and immune evasion mechanisms of *Edwardsiella piscicida* in the largemouth bass (*Micropterus salmoides*). Aquaculture. 2025;604:742505. 10.1016/j.aquaculture.2025.742505.

[60] Yang J, Lin Y, Wei Z, Wu Z, Zhang Q, Hao J, et al. *Edwardsiella ictaluri* almost completely occupies the gut microbiota of fish suffering from enteric septicemia of catfish (Esc). Fishes. 2023;8:30. 10.3390/fishes8010030.

[61] Liang YY, Liu CH. Integrative effects of *Bacillus tropicus* FG2 on growth performance, immunity, gut microbiota, and metabolome in short-finned eel, *Anguilla bicolor pacifica*. Fish Shellfish Immunol. 2026;168:110925. 10.1016/j.fsi.2025.110925. 41076211 10.1016/j.fsi.2025.110925

[62] Guo Z, Chen Y, Wu Y, Zhan S, Wang L, Li L, et al. Changes in meat quality, metabolites and microorganisms of mutton during cold chain storage. Food Res Int. 2024;189:114551. 10.1016/j.foodres.2024.114551. 38876590 10.1016/j.foodres.2024.114551

[63] Ratnawati SE, Kuuliala L, Verschuere N, Cnockaert M, Vandamme P, Devlieghere F. The exploration of dominant spoilage bacteria in blue mussels (*Mytilus edulis*) stored under different modified atmospheres by MALDI-TOF MS in combination with 16S rRNA sequencing. Food Microbiol. 2024;118:104407. 10.1016/j.fm.2023.104407. 38049269 10.1016/j.fm.2023.104407

[64] Ventura M, Perozzi G. Introduction to the special issue “Probiotic bacteria and human gut microbiota”. Genes Nutr. 2011;6:203–4. 10.1007/s12263-011-0241-y. 21779936 10.1007/s12263-011-0241-y PMC3145059

[65] Song MQ, Yu QR, Li EC, Song Y, Cai XY, Huang YX, et al. Leucine improves dietary protein use efficiency by regulating protein synthesis by activating amino acid transporters and the mTORC1 pathways in Chinese mitten crab (*Eriocheir sinensis*). Aquaculture. 2024;581:740423. 10.1016/j.aquaculture.2023.740423.

[66] Xu X, Li X, Xu Z, Yang H, Lin X, Leng X. Replacing fishmeal with cottonseed protein concentrate in practical diet of largemouth bass (*Micropterus salmoides*): growth, flesh quality and metabolomics. Aquaculture. 2024;579:740164. 10.1016/j.aquaculture.2023.740164.

[67] Luan X, Li X, He J, Xu H, Feng W, Chen Q, et al. Effects of arginine family amino acids supplementation on growth, whole-body amino acid profiles, antioxidant capacity, and gene expression of juvenile largemouth bass (*Micropterus salmoides*). Aquaculture. 2025;594:741312. 10.1016/j.aquaculture.2024.741312.

[68] Yu H, Masagounder K, Liang H, Ge X, Huang D, Xue C, et al. DL-methionyl–DL-methionine/DL-methionine supplementation alleviated the adverse effects of dietary low fishmeal levels on growth and intestinal health of *Micropterus salmoides*. Antioxidants. 2024;13:359. 10.3390/antiox13030359. 38539892 10.3390/antiox13030359 PMC10967736

[69] Zhao J, Xu Q. Influence of soybean meal on intestinal mucosa metabolome and effects of adenosine monophosphate-activated protein kinase signaling pathway in mirror carp (*Cyprinus carpio* Songpu). Front Mar Sci. 2022. 10.3389/fmars.2022.844716.

[70] Li X, Zheng S, Wu G. Nutrition and metabolism of glutamate and glutamine in fish. Amino Acids. 2020;52:671–91. 10.1007/s00726-020-02851-2. 32405703 10.1007/s00726-020-02851-2

[71] Zhao J, Yang X, Qiu Z, Zhang R, Xu H, Wang T. Effects of tributyrin and alanyl-glutamine dipeptide on intestinal health of largemouth bass (*Micropterus salmoides*) fed with high soybean meal diet. Front Immunol. 2023. 10.3389/fimmu.2023.1140678. 10.3389/fimmu.2023.1140678 PMC10230952 37266423

[72] Ye J, Lin X, Cheng Z, Su Y, Li W, Ali F, et al. Identification and efficacy of glycine, serine and threonine metabolism in potentiating kanamycin-mediated killing of *Edwardsiella piscicida*. J Proteomics. 2018;183:34–44. 10.1016/j.jprot.2018.05.006. 29753025 10.1016/j.jprot.2018.05.006

[73] Yu Q, Zhang F, Li R, Li E, Qin J, Chen L, et al. Growth performance, antioxidant capacity, intestinal microbiota, and metabolomics analysis of Nile tilapia (*Oreochromis niloticus*) under carbonate alkalinity stress. Aquaculture. 2025;595:741675. 10.1016/j.aquaculture.2024.741675.

[74] Huang X, Ouyang X, Wang L, Xue kun, Yue H, Liao C, et al. Longitudinal analysis of metabolic liver disease induced by high-starch diets in largemouth bass (*Micropterus salmoides*). Aquac Int. 2025. 10.1007/s10499-025-02019-3.

[75] Jia L, Zhang L, Yang H, Li L, Zheng S, Ma Y, et al. Host-intestinal microbiota interactions in *Edwardsiella piscicida-*induced lethal enteritis in big-belly seahorses: novel insights into the role of carbohydrate-active enzymes and host transcriptional responses. Fish Shellfish Immunol. 2025;156:110024. 10.1016/j.fsi.2024.110024. 39557374 10.1016/j.fsi.2024.110024

[76] Wu S, Gao S, Lin D, Bekhit AE-DA, Chen Y. Intestinal barrier restoration in UC: dietary protein/peptide mediate microbiota-Trp-AhR axis and food processing implications. Food Res Int. 2025;217:116799. 10.1016/j.foodres.2025.116799. 10.1016/j.foodres.2025.116799 40597515

[77] Vangaveti VN, Jansen H, Kennedy RL, Malabu UH. Hydroxy octadecadienoic acids: oxidised derivatives of linoleic acid and their role in inflammation associated with metabolic syndrome and cancer. Eur J Pharmacol. 2016;785:70–6. 10.1016/j.ejphar.2015.03.096. 25987423 10.1016/j.ejphar.2015.03.096

[78] Wang Y, Chen Y, Zhang X, Lu Y, Chen H. New insights in intestinal oxidative stress damage and the health intervention effects of nutrients: a review. J Funct Foods. 2020;75:104248. 10.1016/j.jff.2020.104248.

